# Aptamer Development for SARS-CoV-2 and Omicron Variants Using the Spike Protein Receptor Binding Domain as a Potential Diagnostic Tool and Therapeutic Agent

**DOI:** 10.3390/biom15060805

**Published:** 2025-06-01

**Authors:** Prasanna V. Shekar, Anuj Kumar, Nirmitee Mulgaonkar, Samneet Kashyap, Gourav Choudhir, Sandun Fernando, Sachin Rustgi

**Affiliations:** 1 Department of Plant and Environmental Sciences, Clemson University Pee Dee Research and Education Center, Florence, SC 29506, USA; 2 Department of Microbiology and Immunology, Dalhousie University, Halifax, NS B3H 4R2, Canada; 3 Department of Pediatrics, IWK Health Center, Canadian Centre for Vaccinology CCfV, Halifax, NS B3H 4R2, Canada; 4 Biological and Agricultural Engineering Department, Texas A&M University, College Station, TX 77843, USA; 5 Centre for Rural Development & Technology, Indian Institute of Technology (IIT), New Delhi 110016, India; 6 School of Health Research, Clemson University, Clemson, SC 29634, USA; 7 Center for Human Genetics, Clemson University, Greenwood, SC 29646, USA

**Keywords:** *in silico* SELEX (systematic evolution of ligands by exponential enrichment), aptamer, SARS-CoV-2, COVID-19

## Abstract

Despite various methods for detecting and treating SARS-CoV-2, affordable and easily applicable solutions are still needed. Aptamers can potentially fill this gap. Here, we establish a workflow to identify aptamers that bind to the spike proteins of SARS-CoV-2, a process applicable to other targets as well. The spike protein is crucial for the virus’s entry into host cells. The aptamer development process for the spike protein’s receptor binding domain (RBD) begins with splitting the SARS-CoV-2’s genome into 40 nucleotide-long sequences, predicting their two-dimensional structure, and sorting based on the free energy. Selected oligomers undergo three-dimensional structure prediction and docking onto the viral spike protein’s RBD. Six RNA oligomers were identified as top candidates based on the RNA docking with the SARS-CoV-2 wild-type (WT) (Wuhan-Hu-1 strain) and Omicron variant BA.1 RBD and molecular dynamics simulations. Three oligomers also demonstrated strong predicted binding affinity with other SARS-CoV-2 variants, including BA.2, XBB.1.5, and EG.5, based on the protein–aptamer docking followed by stability evaluation using the MD simulations. The aptamer with the best fit for the spike protein RBD was later validated using biolayer interferometry. The process has resulted in identifying a single aptamer from a library of 29,000 RNA oligomers, which exhibited affinity in the submicromolar range and the potential to develop into a viral screen or therapeutic.

## 1. Introduction

Severe acute respiratory syndrome (SARS)-coronavirus (CoV)-2, the causal agent of coronavirus disease 2019 (COVID-19), is a member of the coronavirus family, which consists of enveloped RNA viruses characterized by proteinaceous projections that give them a crown-like appearance, hence the name “coronavirus” [1]. Three different viruses from this family—SARS-CoV-1, Middle East respiratory syndrome (MERS)-CoV, and SARS-CoV-2—have caused severe respiratory disorders in humans in 2002, 2012, and 2019, respectively [2]. While SARS-CoV-1 and SARS-CoV-2 infections led to pandemics, MERS infections remained confined to the Middle East. However, the crude fatality rates of MERS-infected patients were much higher, at 34.4%, compared to SARS-CoV-1, at 9.5%, and SARS-CoV-2, at 2.13%. The COVID-19 pandemic has so far affected 777.7 million individuals worldwide, with 7.1 million deaths as reported by the WHO [3].

**Figure 1:**
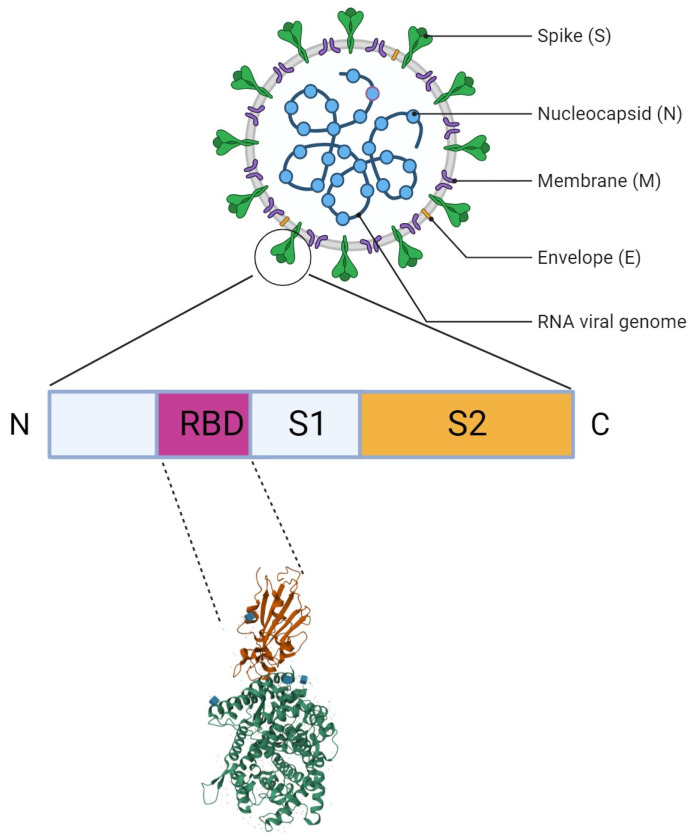
A diagrammatic representation of the SARS-CoV-2 particle, highlighting the location and structure of the RBD complexed with ACE2.

SARS-CoV-2 consists of several structural and nonstructural proteins involved in viral infection and transmission. The structural proteins that form the viral envelope are spike (S), envelope (E), membrane (M), and nucleocapsid (N) [4]. Among these proteins, the S protein is responsible for viral attachment and fusion with the host cell membrane. The S protein, a transmembrane glycoprotein, interacts with the angiotensin-converting enzyme-2 (ACE-2) receptors in the human epithelial and respiratory cells [5]. The S protein is comprised of S1 and S2 subunits. The S1 subunit is involved in the interaction with ACE-2 receptors (adhesion) and the S2 subunit is involved in viral membrane fusion (Figure 1). The S protein is the target for various diagnostic and therapeutic approaches like vaccine development, antibody therapy, or the use of small molecules [6,7].

Aptamers are small, single-stranded (ss) DNA or RNA molecules or peptides that bind to the target with greater affinity and specificity in their tertiary structures [8,9]. They exhibit a wide range of clinical, diagnostic, and therapeutic applications, much like monoclonal antibodies, but unlike them, aptamers are produced *in vitro* [10]. Aptamers offer several advantages over antibodies; for instance, unlike antibodies, aptamers can be raised against toxic and nonimmunogenic targets. Aptamers are ten times smaller than antibodies, allowing them to access targets more efficiently and making them more amenable to chemical modifications. Additionally, aptamers are relatively easy to develop and mass-produce without ethical concerns or batch-to-batch variation, and they are easy to store and transport [11,12]. Due to these attributes, aptamers slowly gained popularity for analytical, imaging, and diagnostic work [13,14,15,16]. Additionally, unlike antisense technology, where the target is intracellular, the aptamer target could be extra- or intracellular. As aptamers primarily bind proteins and often inhibit protein–protein interactions in this process, they elicit therapeutic effects. Indeed, aptamers were used as therapy for macular degeneration and several therapies are under evaluation for hematological disease and cancer [8,15,16].

Numerous approaches have been developed over the years for the aptamer generation. The conventional method for aptamer selection is known as SELEX (systematic evolution of ligands by exponential enrichment). In the case of DNA aptamers, PCR is used to amplify DNA to a desired concentration, and DNA strands are separated by denaturation. The RNA aptamers are generated by *in vitro* transcription of double-stranded (ds) DNA with T7-RNA polymerase. The ssDNA/ssRNA is incubated with the target, and the high-affinity aptamers are separated from the unbound ones. The process is repeated 10–15 times to obtain oligonucleotides with a high affinity for the target [17,18]. However, these methods are time-consuming and expensive [19]. Another relatively inexpensive procedure to achieve a similar goal was proposed in the literature as *in silico* SELEX, and the method was somewhat tested to develop aptamers for various detection and therapeutic purposes [20].

When the pandemic started in 2020, several viral detection methods were developed and approved. Likewise, vaccines were invented and appropriated [21]. These developments occurred at lightning speed. However, in the face of uncertainty related to the pandemic, specifically, its recurrence and emergence of new viral strains (e.g., Omicron strains EG.5 (“Eris”) and BA.2.86 (“Pirola”) in 2023), reliable, faster, and cheaper detection methods are still needed, which can help prevent the spread of the virus, specifically among the unvaccinated. Among several methods developed for viral detection, the quantitative real-time PCR-based test is still the gold standard. Other methods included antibody-based and CRISPR-based tests [22]. Unfortunately, for some tests, the level of reliability is low; for others, the cost is prohibitive or the waiting period for results is too long [23,24].

Aptamer-based detection methods, such as enzyme-linked oligonucleotide assay (ELONA), aptamer-based biosensors (aptasensors), and the aptamer-based lateral flow assay, have been successfully deployed in the past to detect various pathogens [25,26]. A dual biosensor was developed using molecular imprinting polymer and aptamer strings to detect the intact SARS-CoV-2 [27], and another study utilized double-labeled DNA aptamers [28]. However, the aptamers used in these studies were developed using the conventional SELEX method, which is time-consuming and expensive. The development of aptamers using *in silico* methods for detection and diagnostic purposes has proven to be tremendously helpful in biological systems. Studies have shown the use of this approach to design aptamers and screen them for targeting various biomolecules [29,30,31].

This study aims to build an *in silico* aptamer selection pipeline to identify aptamers for the SARS-CoV-2 spike protein, creating an affordable and precise tool for quick clinical diagnosis. This method will enable the identification of new aptamers to meet diagnostic needs as the pathogen continues to spread and evolve, allowing the development of aptamer combinations to effectively detect the viral strains in circulation at any given time. To identify candidate aptamers, the present study used the SARS-CoV-2 genomic RNA sequence and created a library of 40-nucleotide oligomers for an *in silico* SELEX process. Using different criteria during the SELEX process, we identified candidate aptamers and tested them further using bio-layer interferometry (BLI). Due to the importance of spike protein in disease development, it was chosen for aptamer targeting and early detection of viral particles in humans. The pipeline developed in this study could be widely implemented to develop aptamers for other important viral pathogens that can be used for diagnostic and therapeutic purposes.

## 2. Materials and Methods

### 2.1. In Silico Sorting and Selection of Candidate Aptamers

**Figure 2:**
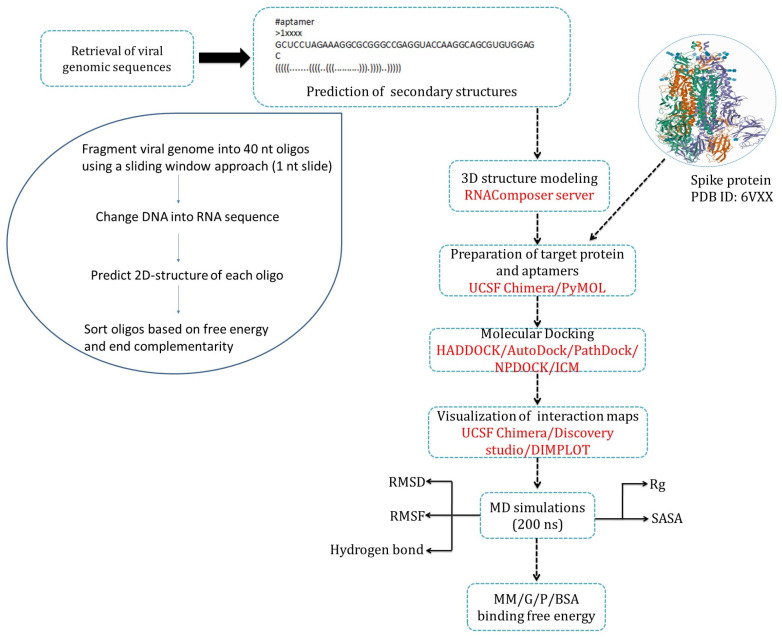
A flowchart of the *in silico* SELEX process.

For the *in silico* SELEX, we retrieved the viral genome sequence (NC_045512.2) from the National Center for Biotechnology (NCBI) (www.ncbi.nlm.nih.gov; accessed on 30 May 2023). Using a Strawberry-Perl-based program with a sliding window approach, the viral genome was split into 40 nucleotide sequences, where each splice differed from the previous one by a single nucleotide. This process generated 40-mer sequences, which were put through the Vienna RNA package to generate corresponding RNA sequences and predict the secondary structure and free energy of each RNA 40-mer. Once the data were processed through the Vienna RNA package, they were sorted in Excel following the parameters described by Chushak et al. [32]: (i) RNA 40-mers with a free energy cutoff of −9.50 kcal/mol and (ii) the secondary structures with fewer than 11 unpaired nucleotides (end complementarity) were removed. Once the data were fully sorted, the sequences that met the criteria were used for 3D structure predictions (Figure 2).

### 2.2. Structural Modeling of RNA Oligomers

The Mfold server (http://www.unafold.org/mfold/applications/rna-folding-form.php; accessed on 30 May 2023) [33] was used to determine the secondary structures of selected RNA oligomers (40-mers). The two-dimensional structures of the RNA molecules were downloaded in Vienna format from the Mfold server output. These 2D structures were then submitted as input to RNAcomposer, a 3D modeling server (https://rnacomposer.cs.put.poznan.pl; accessed on 30 May 2023) [34], for automated RNA structure modeling.

### 2.3. Spike Protein Structure Preparation for SARS-CoV-2 Wild-Type (WT) (Wuhan-Hu-1 Strain) and Mutant Strains

A high-resolution (2.50 Å) protein structure of the spike protein was retrieved from the RCSB Protein Data Bank (PDB ID: 6LZG). This protein was crystalized with ACE2, water, and the ligands NAG and Zn. These molecules were removed from the protein complex using the UCSF Chimera package, version 1.16 (https://www.cgl.ucsf.edu/chimera/; accessed on 30 May 2023). The protein structure was visualized and evaluated for various chemical properties, including hydrogen consistency, bond order, steric clash, and charge, using AutoDock version 1.5.6 (https://autodock.scripps.edu/; accessed on 30 May 2023) and Discovery Studio (https://discover.3ds.com/discovery-studio-visualizer-download; accessed on 30 May 2023). The same protein structure (PDB ID: 6LZG) served as a template for predicting the RBD mutant models of different Omicron variants (BA.1, BA.2, XBB.1.5, and EG.5). These RBD mutant models were manually constructed using the mutagenesis wizard of PyMOL, version 3.1 (https://pymol.org/2/; accessed on 30 May 2023) and UCSF Chimera (https://www.cgl.ucsf.edu/chimera/; accessed on 30 May 2023) was used to validate the dihedral angles of the mutant structures predicted by PyMOL.

### 2.4. Protein and Candidate Aptamer Docking

The selected set of 83 candidate aptamers was subjected to molecular docking against reference RBD and Omicron BA.1 RBD mutant model using the HDOCK server (http://hdock.phys.hust.edu.cn//; accessed on 30 May 2023). After docking with the RBD of reference strain and BA.1, the top-ranked aptamers were shortlisted for further docking with RBD models of three representative Omicron variants, BA.2, XBB.1.5, and EG.5. The HDOCK server works based on the hybrid algorithm of template-based modeling and *ab initio* docking between protein–protein and protein–DNA/RNA [35]. After docking, the candidate aptamers were ranked based on HDOCK score, Root Mean Square Deviation (RMSD) from the overall lowest energy structure, and interaction between a candidate and the Wuhan-Hu-1 Strain or Omicron variant RBD. Docking complexes were rendered in different stereochemical shapes to analyze and annotate the molecular interactions, such as hydrogen bonds and bond lengths, using UCSF Chimera and PyMOL tools. The PDBsum and PLIP (https://plip-tool.biotec.tu-dresden.de; accessed on 30 May 2023) tools were used to demonstrate the interacting residues of each docking complex [36].

### 2.5. Prediction of Aptamer and RBD Complexes Using the AlphaFold3

The top-ranked aptamer candidates (23, 32, and 62) were considered for docking against the RBD models of three representative Omicron variants including BA.2, XBB.1.5, and EG.5, using the state-of-the-art Alphafold3 server (https://alphafoldserver.com/; accessed on 30 May 2023). The PLIP server [36] was employed to predict and visualize the docking complexes predicted using the AlphaFold3.

### 2.6. Molecular Dynamics (MD) Studies

**Figure 3:**
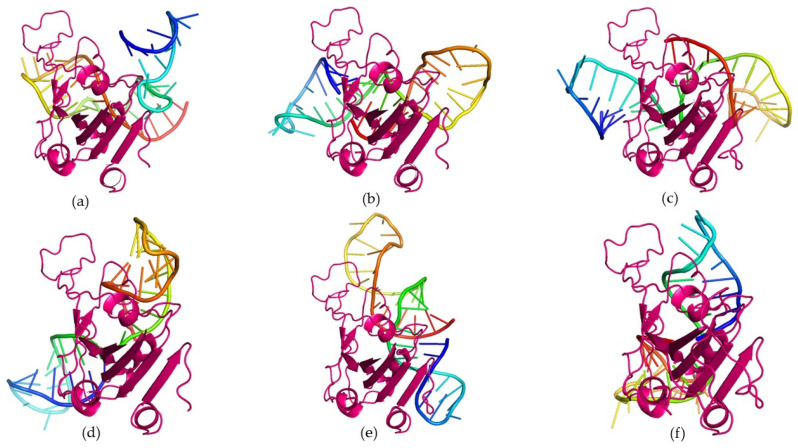
Three-dimensional visualization of docking complexes of top-ranked candidate aptamers and the Omicron BA.1 variant’s spike protein receptor-binding domain (RBD): (**a**) RBD_Aptamer 11 complex; (**b**) RBD_Aptamer 22 complex; (**c**) RBD_Aptamer 23 complex; (**d**) RBD_Aptamer 32 complex; (**e**) RBD_Aptamer 40 complex; and (**f**) RBD_Aptamer 62 complex. In these docking complexes, candidate aptamers are represented by rainbow spectra, while hot pink was used to depict the spike RBD. These complexes were visualized using the PyMOL package.

**Figure 4:**
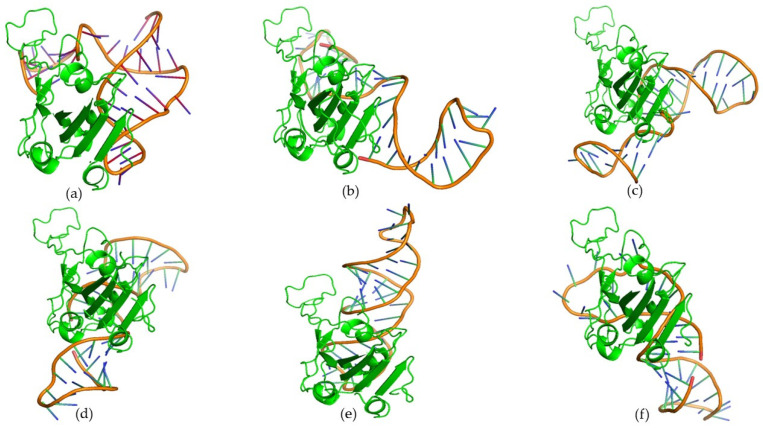
Three-dimensional visualization of docking complexes of top-ranked candidate aptamers and the Wuhan-Hu-1 strain spike protein RBD: (**a**) RBD_Aptamer 11 complex; (**b**) RBD_Aptamer 22 complex; (**c**) RBD_Aptamer 23 complex; (**d**) RBD_Aptamer 32 complex; (**e**) RBD_Aptamer 40 complex; and (**f**) RBD_Aptamer 62 complex. In these docking complexes, candidate aptamers are colored by elements, while green was used to depict spike RBD.

From the docking analysis, we selected a subset of six candidate aptamers, namely, 11, 22, 23, 32, 40, and 62, that were effective against both Wuhan-Hu-1 strain and Omicron RBDs. An MD simulations study was conducted to evaluate the time-dependent stability of the top-ranked docking complexes between the putative aptamers and Wuhan-Hu-1 strain/Omicron RBD (Figure 3 and Figure 4). MD simulation was conducted for 200 nanoseconds using the GROMACS 5.1.1 package following Gajula et al. [37]. The AMBER96 protein, nucleic AMBER94 force field was applied to generate topology for proteins and aptamers [38]. A cubic water box with a TIP3P water model was applied for structure solvation during reactions. The 0.15 M NaCl was added to electro-neutralize the complex structure. The steepest descent algorithm was utilized for structure minimization with 5000 steps. After the energy minimization process, the structure was equilibrated through NVT and NPT ensembles for 5 ns and 10 ns, respectively. Finally, a production run of 200 ns was executed with each step of 2 fs.

### 2.7. Molecular Dynamics Trajectory Analysis

After the completion of the MD simulation run on 200 ns, different quality assurance parameters of MD, namely, root mean square deviation (RMSD), root mean square fluctuations (RMSF), radius of gyration (Rg), solvent-accessible surface (SASA), and number of hydrogen bonds were calculated using the GROMACS package, version 2023.3 by determining conformational flexibility and stability of the MD trajectories. The RMSD and RMSF plots were estimated using gRMS and gRMSF modules, respectively. Other parameters such as Rg, SASA, and hydrogen bonds were calculated using gyrate, gmxsasa, and ghbond built-in commands in GROMACS, respectively.

### 2.8. Binding Free Energy Estimation

The g_mmpbsa [38], a script-based program available in GROMACS, was utilized to calculate the molecular mechanics/Poisson–Boltzmann surface area (MM/PBSA) binding free energy of the complexes processed for MD simulations. Different binding free energy elements (excluding entropic contributions), including binding energy (kJ/mol), van der Waal energy (∆EvdW), electrical energy (∆Elec), polar solvation energy (∆G polar), and SASA, were estimated using the MM/PBSA method as previously described by Kumari et al. [39] and Pathak et al. [40]. In this study, the major elements of the binding free energy of protein–aptamers complexes were analyzed using the following equation

MM/GBSA ΔG_bind_ = ΔG_complex_ − (G_protein_ + G_ligand_)

where G_protein_ reflects the total free energy of the protein, G_ligand_ denotes the total free energy of the ligand in the solvent, and G_complex_ represents the total free energy of the docking complex.

### 2.9. Biolayer Interferometry (BLI)

The top six candidates from the molecular dynamic studies were synthesized from integrated DNA technologies (IDT), Coralville, IA, and analyzed using biolayer interferometry [41]. The lyophilized samples were resuspended and stored as per the manufacturer’s instructions. The BLI experiments were performed in the Octet R4 instrument (Sartorius, Göttingen, Germany) using the Octet BLI Discovery 12.2 Software. Binding experiments were performed in black, flat-bottom 96-well plates (Greiner, Sigma-Aldrich, Inc., St. Louis, MO, United States) at 25 °C using a 1000 rpm orbital shake speed. The his-tagged RBD protein (Sino Biological, Houston, TX, USA) diluted in assay buffer (10 mM phosphate buffer, pH 7.4, 137 mM NaCl, 2.7 mM KCl, 3 mM MgCl_2_, 0.01% BSA, 0.002% Tween 20) at 2.5 µg/mL concentration (loading response ~0.85 nm) was immobilized on HIS1K biosensors (Sartorius, Göttingen, Germany) for 300 s during the loading step. Four 1/2 serial dilutions of aptamer (1400–175 nM) in assay buffer were tested for binding kinetics. Prior to each experiment, the aptamers were diluted in folding buffer (10 mM phosphate buffer, pH 7.4, 137 mM NaCl, 2.7 mM KCl, and 3 mM MgCl_2_) and refolded using a ramp temperature of 1 min at 85–90 °C, 2 min at 65 °C, 2 min at 37 °C, and 3 min at 25 °C. The baseline (60 s), association (60 s), and dissociation (120 s) curves were recorded for each aptamer. Data were corrected using the double reference method: subtraction of the reference well per sample (to negate buffer effects) and reference biosensor (to negate nonspecific binding). The binding sensorgrams were aligned to the baseline step, globally fit to a 1:1 binding model, and analyzed using the Octet Analysis Studio 12.2 Software. Two independent experiments were conducted for each aptamer.

## 3. Results and Discussion

### 3.1. In Silico SELEX Process to Select the Candidate Aptamers

In the present study, instead of screening a conventional RNA oligomer library for aptamers, we fractioned the viral genome into 29,000 oligomers and sequentially reduced the number of undesirable oligonucleotides using different selection criteria in a stepwise fashion. The reasoning behind searching for aptamers within the viral genome, instead of using a library of random oligomers, came from earlier reports that showed parts of the viral genome interact with the nucleocapsid (N) and membrane (M) proteins [42,43]. This suggested that the viral genome could be searched for oligonucleotides with ribonucleoprotein-forming properties. For this analysis, the size of the oligonucleotides was set to 40 nucleotides based on previous experimental studies, where an oligonucleotide library ranging in size from 20–80 nt was found effective in finding aptamers [44]. The first round of selection, based on the free energy cutoff of −9.50 kcal/mol, resulted in a 10-fold reduction in the number of sequences, yielding a set of 2,893 (9.97%) oligonucleotides. The second round of selection, based on the end complementarity of the oligos, further reduced the number by about 3-fold to 83. The elimination of undesirable sequences corresponded with the earlier observations where a rapid enrichment in the more complex potential binders was observed following the abovementioned criteria [45].

### 3.2. Candidate Aptamer Interaction with the SARS-CoV-2 and Omicron (BA.1) RBD

Protein–aptamer docking is a popular molecular modeling approach to screen sequences for candidate aptamers. In the present study, following 3D structure modeling, 83 RNA oligos were docked on the Wuhan-Hu-1 strain and the Omicron variant’s spike protein RBD. The docking results demonstrated that out of 83 candidates, six RNA oligomers, namely, 11, 22, 23, 32, 40, and 62, had low RMSD values, 37.52, 45.85, 24.76, 29.62, 35.89, and 21.24 Å, respectively. Further, these candidates exhibited a binding energy of −325.15, −328.81, −328.46, −318.28, −318.57, and −326.89 kcal/mol, respectively.

**Figure 5:**
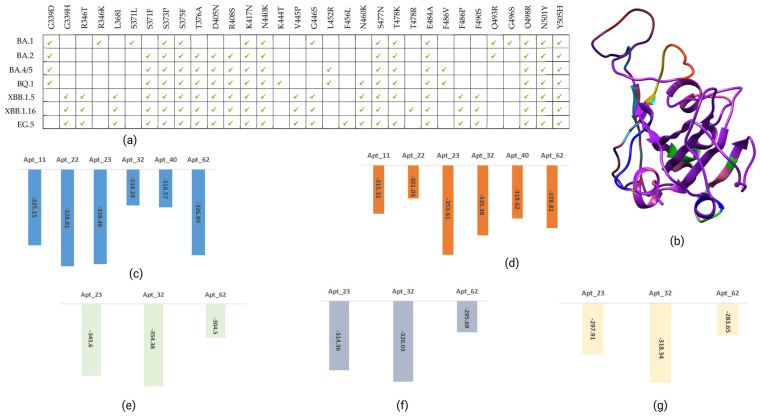
(**a**) Occurrence of common mutations in the RBD of the spike protein across various Omicron variants. These mutations were manually curated based on a literature survey and data from the GISAID Lineage Comparison tool. (**b**) Superimposed 3D structure model of the spike RBD based on the reference SARS-CoV-2 sequence, highlighting the mutant residues observed in emerging Omicron variants: BA.1, BA.2, BA.⅘, BQ.1, XBB.1.5, XBB.1.16, and EG.5. Colors correspond to the following: purple for the reference, hot pink for BA.1, green for BA.2, cyan for BA.⅘, orange-red for BQ.1, blue for XBB.1.5, red for XBB.1.16, and yellow for EG.5. Mutant 3D models of the Omicron variants were manually generated using the mutagenesis wizard in PyMOL (https://pymol.org/2/; accessed on 30 May 2023), with PDB ID_7T9J as a template, and superimposed using the UCSF Chimera package (https://www.rbvi.ucsf.edu/chimera/; accessed on 30 May 2023). (**c**) Calculated binding energies for the top-ranked RNA oligos against the RBD of the Wuhan-Hu-1 strain spike protein. (**d**) Graphical representation of top-ranked oligos against the RBD of the BA.1 strain. (**e**) Representation of calculated binding energies of shortlisted aptamers against the RBD of the BA.2 strain. (**f**) Calculated binding energies of aptamers against the RBD of the XBB.1.5 strain. (**g**) Binding energies of shortlisted aptamers against the RBD of the EG.5 strain.

When docked with the Omicron RBD, the same set of sequences (11, 22, 23, 32, 40, and 62) exhibited low ligand RMSD values, such as 42.05, 40.40, 40.52, 36.29, 31.40, and 46.90 Å, with corresponding binding energies of −315.31, −301.03, −353.61, −335.38, −319.62, and −328.81 kcal/mol, respectively. The RMSD values are predictive of the structural stability of the biomolecules. Given the protein–RNA docking affinity and molecular interaction patterns with the Wuhan-Hu-1 strain and Omicron variant’s spike protein RBD, we ranked 11, 22, 23, 32, 40, and 62 as candidate aptamers (Supplementary Table S1). Furthermore, the prediction and visualization of the interaction plots using PDBSum, PyMOL, and LigPlot^+^ tools revealed that the top-ranked RNA oligos also formed numerous hydrogen bonds and nonbonded contacts with the RBD of the Wuhan-Hu-1 strain and Omicron variant (Figure 5). Two RNA oligos, 32 and 62, were found to manifest a higher number of hydrogen bonds compared to other docking complexes with the Wuhan-Hu-1 strain and the Omicron variant’s spike protein RBD (Supplementary Table S1). As shown in Supplementary Figure S1, the 32_RBD (Wuhan-Hu-1 strain) complex exhibited hydrogen bonds at nine residues (Ser373, Ser375, Trp436, Arg403, Gln409, Asn437, Asn440, Pro499, and Gln506) and fifteen nonbonded contacts (Phe374, Glu406, Arg408, Lys417, Leu441, Tyr453, Phe456, Ser494, Tyr495, Gly496, Tyr508, Asn501, Gly502, Val503, and Tyr505). Likewise, nine residues of the Wuhan-Hu-1 strain spike protein RBD—Arg408, Tyr421, Lys417, Asn437, Tyr473, Thr376, Gln498, Tyr449, and Tyr508—interacted with the aptamer 62 via hydrogen bonds and sixteen nonbonded contacts (Ser375, Arg403, Asp405, Thr415, Gly446, Phe456, Arg457, Ala475, Gly485, Phe486, Tyr489, Ser494, Val503, Tyr505, Gln506, and Tyr508).

As shown in Supplementary Figure S1, in the 32_RBD (Omicron) complex, eleven residues formed hydrogen bonds (Arg403, Thr415, Gly416, Asp420, Tyr421, Ser446, Tyr473, Asn487, Ser496, Thr500 and His505) and an equal number of residues exhibited nonbond contacts (Asn417, Lys424, Asp427, Val445, Tyr449, Phe456, Asn460, Leu461, Tyr489, Arg493 and Tyr501). In the 62_RBD (Omicron) complex, nine residues (Arg403, Arg408, Tyr421, Ala475, Asn487, Tyr489, Ser496, Gln498, and Thr500) formed hydrogen bonds, while six residues (Asp405, Asn477, Lys458, Tyr473, Tyr501, and Gly502) interacted through nonbond contacts (Supplementary Table S1). The binding affinities and interaction patterns observed in this study are consistent with earlier studies that screened aptamers as potential inhibitors of SARS-CoV-2 spike protein and its variants [29,31,46].

Notably, the docking and molecular interaction studies revealed that top-ranked oligos also form numerous hydrogen bonds and nonbond contacts with Omicron spike RBD containing the G446S, G496S, Q498R, K417N, E484A, Q493R, N501Y, and Y505H mutations. This strong binding pattern with the mutant residues indicates strengthened interactions, showing a tight fit to the Omicron spike RBD. These key mutations targeted by top-ranked oligos are well known to play an important role in antibody resistance and infectivity of SARS-CoV-2 and its variants [20,47,48,49]. It was shown previously that a combination of mutations like Q498R, N501Y, and Y505H epistatically enhances ACE2 interaction [50]. Taken together, the docking results revealed that the top-ranking oligos identified in this study have the potential to inhibit the ACE2-RBD interface of the Wuhan-Hu-1 strain and the Omicron (B.A.1) variants.

### 3.3. Interactions Between the Top-Ranked Candidate Aptamers and RBD of Representative Omicron Variants (BA.2, XBB.1.5, and EG.5)

Based on the docking results with the RBD of the Wuhan-Hu-1 strain and BA.1 and considering their binding affinity and the number of hydrogen bonds with mutant residues in the Omicron BA.1 RBD structural model, three RNA oligomers (23, 32, and 62) were selected as top-ranked candidates. As shown in Figure 5, emerging Omicron variants share similar mutation patterns within the RBD, with only a few unique mutations. Therefore, these shortlisted aptamers were further evaluated for their binding affinity to three representative Omicron variants (BA.2, XBB.1.5, and EG.5).

#### 3.3.1. Interaction Patterns of Shortlisted Candidate Aptamers with RBD of the BA.2 Spike Protein

The protein-RNA docking results revealed that RNA oligomers 23, 32, and 62 were found to have binding energies of −343.60, −354.38, and −304.50 kcal/mol, respectively, with lower RMSD values of 0.70, 2.79, and 21.86 Å. The 23_RBD complex showed hydrogen bonds with six residues: Thr415, Asn417, Tyr449, Arg493, Ser494, and Tyr501. Out of these six hydrogen bonds, three interactions were noted with the mutant residues (Supplementary Table S1). RNA oligomer 23 was found to have nonbonded interactions with ten residues, including a mutant residue, His505. After exploring the interaction patterns, it was noted that the 32_RBD complex formed hydrogen bonds with seven residues: Arg403, Gly416, Asp420, Tyr421, Tyr449, Tyr473, Asn487, and a total of eleven nonbonded interactions, including two mutant residues (Arg493 and Arg498). In the case of the 62_RBD complex exhibited hydrogen bond interactions at nine residues, Arg403, Tyr421, Tyr449, Tyr489, Arg493, Arg498, Thr500, Tyr501, His505. Among these residues, four were mutant residues in the BA.2 spike protein RBD (Supplementary Table S1). The residues Asn477, Phe456, Ser494, and Gly496 interacted via nonbonded interactions. The 62_RBD complex was found to have the maximum number of hydrogen bond interactions with mutant residues in the BA.2 spike protein RBD.

#### 3.3.2. Shortlisted Candidate Aptamer Interactions with the RBD of XBB.1.5 Spike Protein

The docking results demonstrated that aptamers 23, 32, and 62 were found to have binding energies of −314.36, −320.03, and −295.69 kcal/mol, respectively. Further, these candidates showed lower RMSD values of 57.76, 29.64, and 22.48 Å, respectively. The docking complex of 23_RBD was stabilized with five hydrogen bonds involving Thr333, Pro337, His339, Lys440, and Asn343 residues. Among these hydrogen-bond-forming residues, two-His339 and -Lys440 were mutant residues in the RBD of XBB.1.5 spike protein. Ten residues provided stability to the complex with nonbonded interactions. Among these ten nonbonded interactions, two involved mutant residues Phe371 and Phe375. The 32_RBD complex involved the formation of a hydrogen bond with one mutant residue, Ser490, and three other residues (Tyr449, Gly482, and Gln493), which provided stability to the conformation. This complex was found to have a total of eleven nonbonded interactions, including interactions with two mutant residues (Ala484 and Tyr501) (Supplementary Table S1). As shown in Supplementary Table S1, the aptamer 62 forms hydrogen bonds with two mutant residues Arg498 and Tyr501 of the XBB.1.5 spike protein RBD and four hydrogen bonds with other residues (Tyr449, Tyr489, Gly496, and Thr500). This docking complex also manifested a total of six nonbonded interactions (including mutant residues Asn477 and His505 and other residues Tyr449, Tyr489, Gly496, and Thr500).

#### 3.3.3. Shortlisted Candidate Aptamer Interactions with the RBD of EG.5 Spike Protein

The shortlisted RNA oligomers 23, 32, and 62 exhibited low RMSD values of 38.53, 29.37, and 34.79 Å, with corresponding binding energies of −297.91, −318.34, and −283.65 kcal/mol, respectively, indicating strong interaction patterns with the RBD of the Omicron EG.5 spike protein. The aptamer candidate 23_RBD complex exhibited hydrogen bond interactions with five residues (Arg403, Asn405, Gly485, Ser490, and Arg498), among which Asn405, Ser490, and Arg498 are mutated residues in the EG.5 Spike protein. Nonbonded interactions with mutant residues (Ser446, Ala484, Pro486, Arg498, and Tyr501, His505) and other residues (Tyr449, Val483, Tyr489, and Gln493) were also observed (Supplementary Table S1). After exploring the interaction map of the 32_RBD complex, it was observed that four residues (Arg403, Tyr449, Ser490, and Gln493) manifested hydrogen bonds, with Ser490 being a mutant residue. The complex of 32_RBD also stabilized with nine nonbonded contacts, including an interaction with the mutant residue Tyr501. In the case of the 62_RBD complex, a total of six residues (Lys378, Cys379, Gly381, Ser383, Gln414, and Asp427) established hydrogen bond interactions. The mutant residues (Phe375, Asn405, and Tyr501) and other residues of RBD (Phe377, Tyr380, Val382, Pro384, Val503, Gly504) showed the formation of nonbonded contacts while interacting with oligo 62. Among these, aptamer 23 demonstrated a maximum number of hydrogen bonds and nonbonded contacts with mutant residues in the RBD of the EG.5 spike protein.

### 3.4. Top-Ranked Aptamers Interactions with the RBD of Representative BA.5, EG.5, and XBB.1.5 Variants Using AlphaFold 3 (AF3)

**Figure 6:**
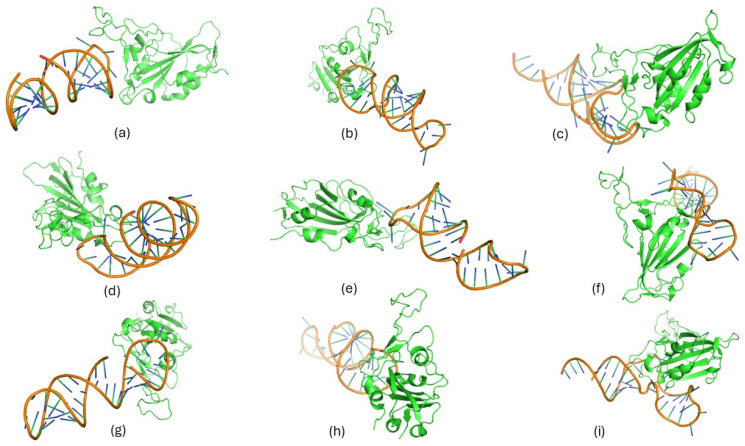
Three-dimensional visualization of docking complexes of RBD from the BA.2, EG.5, and XBB.1.5 variants and top-ranked candidate aptamers (23, 32, 62), predicted using the AF3. (**a**) BA.2 RBD_Aptamer 23, (**b**) BA.2 RBD_Aptamer 32, (**c**) BA.2 RBD_Aptamer 62, (**d**) EG.5 RBD_Aptamer 23, (**e**) EG.5 RBD_Aptamer 32, (**f**) EG.5 RBD_Aptamer 62, (**g**) XBB1.5 RBD_Aptamer 23, (**h**) XBB1.5 RBD_Aptamer 32, and (**i**) XBB1.5 RBD_Aptamer 62. The green ribbon structure represents the RBD, while aptamers are represented with an orange helix. These complexes were visualized using PyMOL.

**Figure 7:**
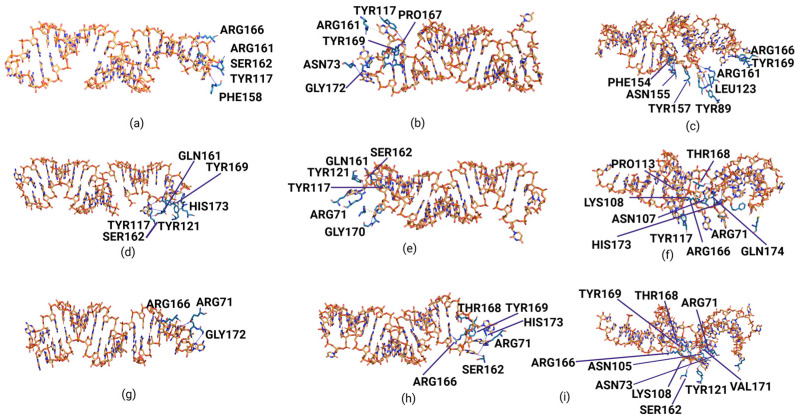
Interaction visualization for the docking complexes of RBD from the BA.2, EG.5, and XBB.1.5 variants and top-ranked candidate aptamers (23, 32, 62), visualized using the PLIP server (**a**) BA.2 RBD_Aptamer 23, (**b**) BA.2 RBD_Aptamer 32, (**c**) BA.2 RBD_Aptamer 62, (**d**) EG.5 RBD_Aptamer 23, (**e**) EG.5 RBD_Aptamer 32, (**f**) EG.5 RBD_Aptamer 62, (**g**) XBB1.5 RBD_Aptamer 23, (**h**) XBB1.5 RBD_Aptamer 32, and (**i**) XBB1.5 RBD_Aptamer 62. The interacting amino acids from RBD are shown in rainbow colors, while aptamers are represented with an orange helix. Interacted residues are labelled with the gray color, while different colorful lines represent different types of interaction bonds.

Three top-ranked aptamers—23, 32, and 62—targeting the RBDs of the representative BA.5, EG.5, and XBB.1.5 variants were docked using Google DeepMind’s AF3 server. The docked complexes of RBDs and aptamers showed stable interaction patterns, with pTM scores ranging from 0.74 to 0.8, while the ipTM scores ranged from 0.26 to 0.51. Compared to other complexes, two docking complexes, namely 32_RBD and 62_RBD of the BA.2 variant, showed the highest pTM scores as 0.8. Per the general rule, a pTM score higher than 0.5 is always considered a good score. In the present study, all docked complexes showed a pTM score higher than 0.5, which strongly indicates the accuracy of predicted complexes with higher confidence and interaction interface reliability between docked RBD and aptamers. Predicted complexes also demonstrated a strong interaction pattern and were found to have a set of interactions, including hydrogen bonds, hydrophobic interactions, salt bridges, π-stacking, and π-cation interactions. Based on the predicted interactions, it was observed that complexes of 32_RBD BA.2, 62_RBD BA.2, 62_RBD EG.5, and 62_RBD XBB.1.5 showed the highest number of hydrogen bonds compared to other docked complexes (Figure 6 and Figure 7). A list of the hydrogen bonds and other types of interactions predicted for docked complexes of the top-ranked aptamers (23, 32, and 62) and RBD of the representative variants, BA.2, EG.5, and XBB.1.5, is presented in Table 1. Taken together, predicted docked complexes using the revolutionary AF3 tool exhibited a strong interaction pattern between RBD and aptamers with evidence of several hydrogen bonds and other types of chemical interactions.

Based on the comparative interaction analysis of the RBD–aptamer complexes performed using the two popular servers, HDOCK and AF3, it was observed that the outcomes of these tools shared several similarities and disparities. As shown in Figure 3, Figure 4, Figure 6 and Figure 7, the docking complexes predicted using the HDOCK showed the RBD interaction with both paired and unpaired bases, while complexes predicted using the AF3 algorithm demonstrated a dominant interaction pattern with the RNA loop. Complexes predicted using both algorithms showed several molecular interactions. For instance, the 32_RBD Wuhan-Hu-1 and 32_RBD Omicron strain complexes, predicted using HDOCK, exhibited a higher number of hydrogen bonds—9 and 11, respectively—compared to other complexes (Supplementary Figure S1). In contrast, predictions from AF3 indicated a greater number of hydrogen bonds in the 62_XBB.1.5 RBD (*n* = 12) and 62_EG.5 RBD (*n* = 10) complexes (Table 1). Notably, compared to AF3-generated complexes, those predicted using HDOCK exhibited stronger molecular interactions with the mutative residues of the RBD (Figure 3 and Figure 4). Taken together, both docking algorithms demonstrated favorable interaction patterns between the RBD proteins of different variants and the shortlisted aptamers.

### 3.5. Molecular Dynamics Simulations

For this, 200 ns state-of-the-art MD simulations were employed to evaluate the stability of the top-ranked complexes using AMBER96 protein and nucleic AMBER94 forcefield parameters embedded in the GROMACS package.

#### 3.5.1. Root Mean Square Deviation (RMSD)

**Figure 8:**
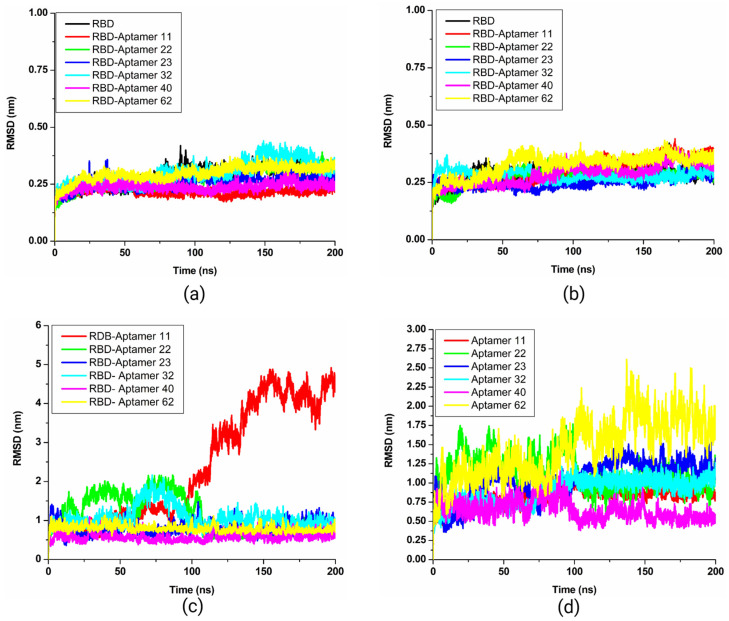
Plots showing calculated backbone and ligand root mean square deviation (RMSD) values; (**a**) the Wuhan-Hu-1 strain spike protein RBD backbone RMSD; (**b**) the Omicron variant spike protein RBD backbone RMSD; (**c**) the Wuhan-Hu-1 strain spike protein RBD backbone ligand RMSD; and (**d**) the Omicron variant spike protein RBD backbone ligand RMSD.

In the calculated backbone, the RMSD plot of the SARS-CoV-2 spike RBD showed constant stability throughout the interaction within a calculated range of ~0.25–0.50 nm. An RMSD plot showed the stable conformation of five (11, 22, 23, 40, and 62) out of six candidate aptamers between 25 ns to 200 ns on ~0.25 nm (Figure 8a). These five aptamers shared an almost similar trend of stability with small conformational changes in RMSD values, except candidate 32, which showed a significant fluctuation between 25 and 150 ns up to ~4 nm. Precisely, between 25 and 80 ns, candidate 32 showed fluctuation. After 80 ns, the candidate showed stability up to 125 ns. In the second wave, fluctuation was observed between 125 and 155 ns, and after that, the candidate showed stability until 200 ns. The RMSD plot of the Omicron spike RBD also showed an almost similar trend of stability throughout the simulations without any significant fluctuations (Figure 8b). The calculated RMSD backbone plots for the Wuhan-Hu-1 strain and the Omicron spike RBD suggested that all complexes showed a relatively smaller number of conformation fluctuations compared to the control molecule.

The measured ligand RMSD plot of the Wuhan-Hu-1 strain spike RBD in complex with top-ranked candidate aptamers exhibited the RMSD range between ~0.5 to ~5.1 nm. Except for RBD_11 (red), all candidates attained stability throughout 200 ns simulations. As evident from Figure 8c, the RBD_11 (red) complex showed a major fluctuation after ~100 ns. Two other complexes, RBD_22 (green) and RBD_32 (cyan), exhibited a small number of fluctuations initiating from ~100 ns on ~1.8 nm. After ~100 ns, these aptamers showed a similar trend of stability up to 200 ns. There was no significant effect of these fluctuations found on the backbone. In the case of calculated ligand RMSD plots of Omicron, the RBD_62 complex (yellow) exhibited higher fluctuations throughout the simulation (Figure 8d). The complex of RBD_22 (green) also showed few fluctuations up to 100 ns; however, after 100 ns, this complex showed a stability pattern. The calculated RMSD plots showed a stronger stability pattern than the RMSDs reported in previous studies for aptamer–spike complexes [51,52,53,54].

#### 3.5.2. Root Mean Square Fluctuation (RMSF)

**Figure 9:**
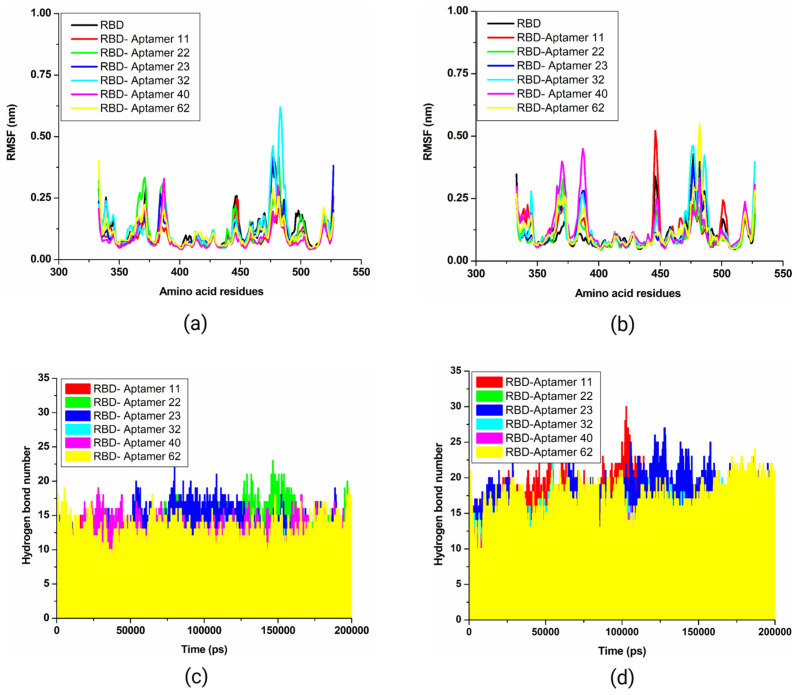
Plots showing root mean square fluctuations (RMSF) and hydrogen bonds: (**a**) the Wuhan-Hu-1 strain spike protein RBD RMSF, (**b**) the Omicron variant spike protein RBD backbone RMSF, (**c**) the Wuhan-Hu-1 strain spike protein RBD number of hydrogen bonds, and (**d**) the Omicron variant spike protein RBD backbone hydrogen bonds.

The root mean square fluctuation (RMSF) plot was calculated with the primary objective of determining the individual residue flexibility of the system over 200 ns. The measured RMSF plot of the Wuhan-Hu-1 strain spike RBD reflected an almost similar pattern of fluctuation across the entire structure throughout the simulation. However, candidate 32 showed a fluctuation between 465 to 500 amino acid residues. RMSF plot values of five candidate aptamers, 11, 22, 23, 40, and 62, demonstrated relatively less conformational fluctuation than the control (Figure 9a). In Figure 9b, the Omicron spike RBD RMSF plots for complexes RBD_62 (yellow) and RBD_11 (red) exhibited the highest peaks between 450 to 500 residues on ~0.50 nm when compared with other simulated systems. Two peaks were also observed in the RBD_40 (pink) complex plot between 350 and 400 residues. Farouk et al. [55] previously reported the same pattern of fluctuations for the Wuhan-Hu-1 strain spike RBD between 365 and 385 residues, 415 and 435 residues, and the loop region residues between 477 and 485. Peaks observed in the present study also belong to the same regions and are consistent with previous reports [55,56]. Altogether, calculated RMSF plots of docking complexes suggested that the spike RBD of the Wuhan-Hu-1 strain and Omicron interact significantly with candidates 11, 22, 23, 40, and 62.

#### 3.5.3. Hydrogen Bonds in Docking Complexes

The presence of hydrogen bonds was predicted for docking complexes between aptamers and spike protein RBD of the Wuhan-Hu-1 strain and Omicron variants throughout the simulation period of 200 ns. The hydrogen bond plot of the Wuhan-Hu-1 strain spike RBD estimated 10 to 24 hydrogen bonds distributed throughout the simulation, with bond lengths ranging from 0.25 and 0.35 nm. RBD_22 (green) and RBD_23 (blue) complexes showed the highest number of hydrogen bonds between ~75 ns to ~150 ns (Figure 9c). In comparison, the Omicron spike RBD complexes with six candidate aptamers demonstrated a higher number of hydrogen bonds than the complexes of these oligos with the Wuhan-Hu-1 strain spike RBD. As shown in Figure 9d, candidates 11, 22, 23, 32, 40, and 62 indicated sustained hydrogen bonding with the Omicron spike RBD. Each of these six candidates was found to have an average of ~13 ± 3, ~9 ± 2, ~14 ± 3, 10 ± 2, 8 ± 2, and 13 ± 2 hydrogen bonds, respectively, as shown in Table 2.

Around 100 ns, the RBD_11 (red) complex showed the highest number of hydrogen bonds (Figure 9d). Previously, Lin et al. [52] reported that the number of hydrogen bonds between spike RBD and aptamers ranged from the highest 10 to the lowest 0 in MD simulations on a time scale. In another similar study [57], authors evaluated the pattern of hydrogen bonds between the designed MEZ aptamer and the RBDs of both Wuhan and Omicron strains using MD simulations on a 150 ns time scale. The study reported a significant difference in the pattern of hydrogen bonds. The complex of MEZ and Omicron strain RBD was found to have a smaller number of hydrogen bonds compared to the complex of MEZ and Wuhan-Hu-1 strain RBD.

The present study calculated more hydrogen bonds than previous reports on aptamers developed against the spike RBD of Wuhan-Hu-1 and Omicron strains [29,31]. According to the present understanding, more hydrogen bonds are considered vital for the strong binding of ligands or aptamers to the pockets of the receptor molecule [58,59]. Therefore, the significant distribution of hydrogen bonds suggested that aptamers could interact with the spike RBD of the Wuhan-Hu-1 strain and Omicron variants and maintain the stability of the interaction complex.

#### 3.5.4. Evaluation of Complexes Using Rg and SASA

**Figure 10:**
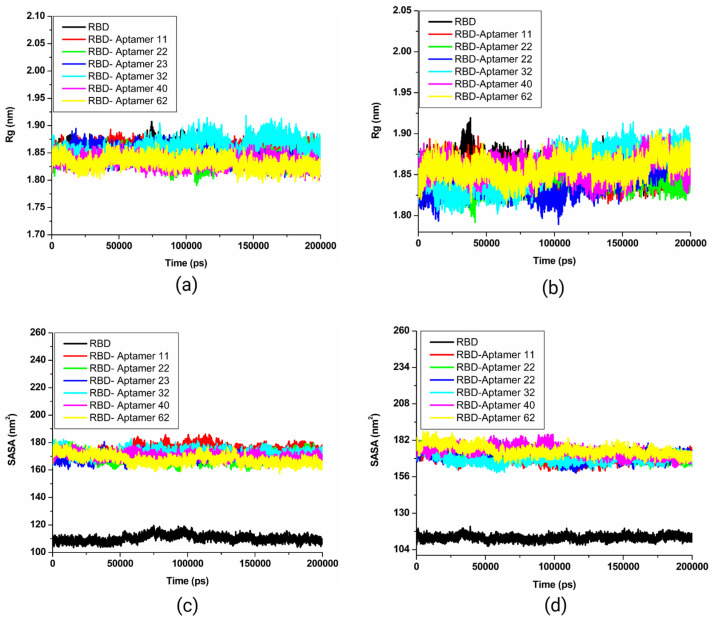
Rg and SASA plots. (**a**) The Wuhan-Hu-1 strain spike protein RBD Rg, (**b**) the Omicron variant spike protein RBD backbone Rg, (**c**) the Wuhan-Hu-1 strain spike protein RBD SASA, and (**d**) the Omicron variant spike protein RBD backbone SASA.

The radius of gyration (Rg) and solvent-accessible surface area (SASA) plots were respectively produced to determine the compactness of the interaction complex over time and to measure the receptor exposure to the solvents. The calculated Rg values for the Wuhan-Hu-1 strain spike RBD docked complexes with six candidate aptamers, 11, 22, 23, 32, 40, and 62, were between 1.8 to 1.95 nm during 200 ns simulations (Supplementary Table S1). As shown in Figure 10a, all six docking complexes of the Wuhan-Hu-1 strain spike RBD and candidate aptamers showed a consistent pattern of stability throughout the simulations without any significant fluctuations. The calculated Rg plot of the Omicron spike RBD docked with candidate aptamers revealed that up to 100 ns, all complexes, along with the control, showed minimal fluctuations. Whereas after 100 ns, all complexes exhibited a straight line without significant fluctuations, reflecting minimum conformational changes (Figure 10b). As illustrated in Figure 3b, the Rg values of the control and docking complexes are noted between ~1.80 and ~1.91 nm throughout the 200 ns simulation. The calculated Rg plots indicated that the binding of candidate aptamers to the spike RBD of the Wuhan-Hu-1 strain and Omicron variants does not change over the simulation period.

The calculated SASA values for complexes of the Wuhan-Hu-1 strain spike RBD and candidate aptamers ranged from 160 and 180 nm^2^, while the complexes of the Omicron spike RBD and candidates ranged from 160 to 182 nm^2^. The SASA values were higher in both cases than the control system SASA values (Figure 10c,d). These observations indicate that the complexes of aptamers and spike RBD have a large surface area exposed to solvent molecules compared to the control system. All complexes and control systems displayed a steady trajectory in both plots without significant fluctuations throughout the 200 ns simulation. Low fluctuations in SASA analysis during MD simulations are generally good for the stability of the docking complexes, while higher SASA values indicate the expansion of protein volume, which can lead to instability [58]. Taken together, the calculated SASA plots suggest that the candidate aptamers’ binding does not significantly affect protein folding.

#### 3.5.5. Estimation of Binding Free Energy

MM/PBSA is a widely utilized method for calculating the ligand binding affinities in protein systems. In this study, we employed this well-established method to estimate the binding free energy of candidate aptamer–RBD complexes. The average binding free energy of six top-ranked complexes (11_RBD, 22_RBD, 23_RBD, 32_RBD, 40_RBD, and 62_RBD) between RNA oligos and Omicron spike RBD was also determined. The binding free energy is a compilation of different energies, including ∆EvdW (kJ/mol), ∆Elec (kJ/mol), ∆G polar (kJ/mol), and SASA (kJ/mol). These energies elaborate on the interactions between selected RNA oligos and the Omicron spike protein RBD at the atomic level. As mentioned in Table 2, the estimated binding free energy of candidate aptamers is as follows: 11, −4653.091 ± 170.939 kJ/mol; 22, −4899.591 ± 207.097 kJ/mol; 23, −5198.452 ± 263.879 kJ/mol; 32, −5439.458 ± 176.559 kJ/mol; 40, −4796.462 ± 134.095 kJ/mol; and 62, −5204.824 ± 156.228 kJ/mol. As per the general understanding, negative binding energy values indicate a significant interaction between RNA oligos and the receptor protein [58,60]. Among candidates, 32 (−5439.458 ± 176.559 kJ/mol) exhibited the maximum negative binding energy, and 62 (−5204.824 ± 156.228 kJ/mol) the second smallest value. In a previous study [61], the binding affinity of the Spike-ACE2 complex was reported as −57.6 ± 0.25 kcal/mol (ΔGMMPBSA), which is higher than the binding affinity of the aptamers against the RBD protein calculated in the present study. The high negative binding energy values observed here may be influenced by van der Waals and electrostatic interactions, which are known to significantly contribute to duplex binding energy [62].

The binding free energy estimations were consistent with the docking results, in which candidates 32 and 62 formed a higher number of hydrogen bonds with the spike protein RBDs of both the Wuhan-Hu-1 strain and the Omicron variant.

### 3.6. Biolayer Interferometry-Based Validation of the Candidate Aptamers

**Figure 11:**
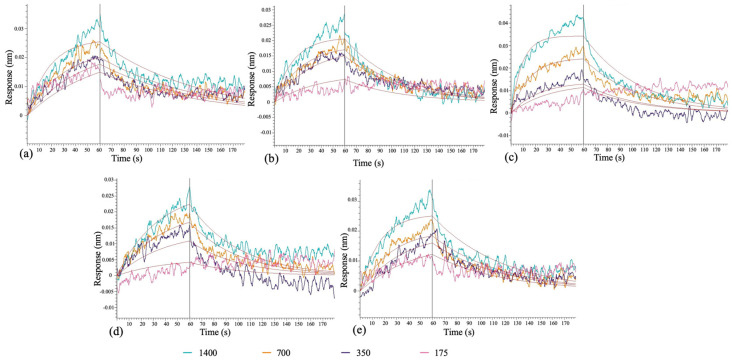
The plots show binding sensorgrams for candidate aptamers 62 (**a**), 40 (**b**), 23 (**c**), 22 (**d**), and 11 (**e**). Two independent experiments (*n* = 2) were conducted for all aptamers, and representative sensorgrams are presented.

Based on molecular docking and MD simulations results, five candidate aptamers 11, 22, 23, 40, and 62 were selected for synthesis and validation of the *in silico* SELEX process results using Biolayer interferometry. To determine the binding of candidates to the Wuhan-Hu-1 strain spike protein RBD, a His-tagged version of it was immobilized onto HIS1K biosensors coated with anti-his antibody, and the binding kinetics were evaluated in real-time for various candidate aptamer concentrations. In the representative binding sensorgrams, as shown in Figure 11, all aptamers demonstrated a dose-dependent binding response to RBD.

Furthermore, the binding association and dissociation rate constants, ka and kd, respectively, and the affinity constant (KD) were calculated by fitting the association and dissociation curves to a 1:1 binding model. The results indicate that 11, 40, and 62 exhibited stronger binding affinities of 0.425 ± 0.146 µM, 0.571 ± 0.173 µM, 0.285 ± 0.141 µM, respectively, to RBD. Comparatively, the other two candidates, 22 and 23, showed weaker binding affinities of 5.45 ± 1.70 µM and 29.3 ± 10.4 µM, respectively. Further, 11 and 62 showed faster association rates (4.29 ± 1.47 × 10^4^, and 4.50 ± 1.89 × 10^4^ M^−1^ s^−1^) compared to 22, 23, and 40 (42.7 ± 8.61 × 10^2^, 9.62 ± 2.05 × 10^2^, and 36.2 ± 7.86 × 10^3^ M^−1^ s^−1^). However, similar dissociation rates were observed for all candidate aptamers. The kinetic constant data are provided in Table 3.

In the BLI experiments, all aptamers exhibited an ability to bind to the spike RBD, with the strongest affinity reported for candidate 62 in the sub-micromolar range. An increased binding response was observed for increasing candidate aptamer concentrations, a trend shown by all candidates. These results are encouraging. Previous studies have reported DNA aptamers that specifically bind to the RBD of spike protein (Wuhan-Hu-1 strain) with affinities in the nanomolar range [63]. Valero et al. [64] reported an RNA aptamer RBD-PB6 (80 nt) and its truncated version (53 nt) that exhibited nanomolar affinities to the RBD. Further truncation and shortening of the aptamer resulted in a complete loss of binding affinity. Further evaluation of the trimerized and more complex versions of the same aptamer enhanced the binding affinities to the lower picomolar range. On the contrary, the aptamers reported in this study are shorter (40 nt) and less complex in structure, making them favorable for therapeutic purposes [52,65].

Experimental techniques like BLI measure binding affinities under specific laboratory conditions, directly observing the interactions between molecules. These methods provide empirical data that can serve as a benchmark for evaluating the accuracy of computational predictions. The significant differences in binding energies between our computational results and those obtained experimentally can be attributed to methodological variations and the inherent differences in measurement scales between computational modeling and experimental assays.

The binding energies reported in our study are derived from predictions for protein-aptamer interactions using the HDOCK server. This platform utilizes a hybrid algorithm that integrates template-based modeling with *ab initio* free docking. Such an approach can produce binding energy values that are significantly higher than those typically observed in experimental settings. This discrepancy arises because computational models, including those generated by HDOCK, may not fully replicate the complex physical and chemical environments influencing molecular interactions *in vitro* or *in vivo*. Furthermore, the binding energy calculations from molecular dynamics (MD) simulations in this study incorporate various energy components, such as van der Waals forces, electrostatic interactions, solvation energies, and the solvent-accessible surface area (SASA). Each of these factors contributes to the overall binding energy estimation, which, in a computational context, can markedly differ from experimental measurements. Our results showed more negative free energy compared to previously reported total energies [31]. Per the general rules of docking and MD simulations, more negative energies are the reflection of the stability of the complexes at the atomic level [51,52]. Based on that, we can conclude the docked complexes reported in the present study are significantly stable.

### 3.7. Protocol Validation Using Aptamers Reported Earlier

**Figure 12:**
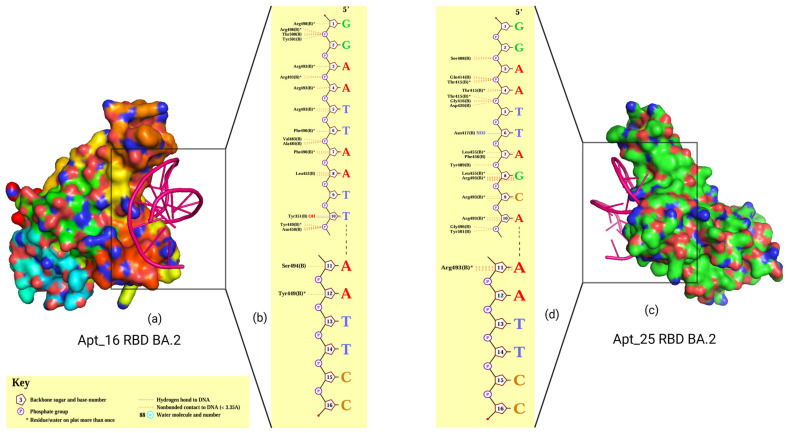
Docking of apt16 and apt25 [46] on the RBD of the BA.2 spike protein: (**a**) 3D representation of the apt16-RBD BA.2 complex; (**b**) interaction plot of apt16-RBD BA.2 complex; (**c**) 3D representation of apt25-RBD BA.2 complex; and (**d**) interaction plot of apt25-RBD BA.2 complex. The BA.2 RBD is shown in a surface representation, while the aptamers are depicted in pink using a cartoon representation. Complexes were rendered with the PyMOL and PDBSum programs.

To validate our aptamer screening protocol, we used two previously reported aptamers, apt16 and apt25 [46]. Both aptamers were docked onto the mutative RBD model of the representative BA.2 variant using the HDOCK server (http://hdock.phys.hust.edu.cn//; accessed on 30 May 2023), followed by interaction analysis. Protein–aptamer docking revealed a stable binding pattern between the BA.2 RBD and both aptamers: apt16 (docking score: −249.97; ligand RMSD: 54.77) and apt 25 (docking score: −248.64; ligand RMSD: 39.60). Interaction analysis showed that apt16 and apt 25 each demonstrated as many as 17 nonbonded contact interactions (<3.35 Å) and formed one hydrogen bond. Apt16 and apt 25 exhibited hydrogen bond interactions with Tyr351 and Asn417, respectively (Figure 12).

When comparing the interaction patterns of the aptamers screened in this study with those previously reported [46], we observed that the top-ranked aptamers in this study formed a greater number of hydrogen bond interactions, indicating more stable docking conformations of the RBD–aptamer complexes. In conclusion, these findings validate our screening protocol and support its use in future studies.

## 4. Conclusions

Early detection of SARS-CoV-2 is crucial for effective disease management. As the virus mutates rapidly, it generates numerous new variants, necessitating rapid, specific, and cost-effective diagnostic tests. The current diagnostic method, RT-PCR, is both expensive and time-consuming. One potential solution is the use of aptamers for detection, which are equally sensitive, adaptable to pathogen mutations, scalable, and more economical than other methods. Aptamers enable rapid virus detection without extensive laboratory resources. However, developing aptamers using the conventional SELEX process is lengthy. This study utilized an *in silico* SELEX process for aptamer selection, incorporating methods such as molecular docking and MD simulations. Six candidate aptamers were identified through these advanced methods, with results suggesting that these candidates (11, 22, 23, 32, 40, and 62) strongly interact with the spike protein RBD of the Wuhan-Hu-1 strain and Omicron variant BA.1, as evidenced by atomic-level evaluation through 200 ns MD simulations. Additionally, three representative candidate aptamers (23, 32, and 62) were evaluated for their binding patterns with other emerging SARS-CoV-2 variants (BA.2, XBB.1.5, and EG.5). All exhibited strong interactions, suggesting their ability to detect these variants, supported by negative binding affinities and the number of hydrogen and nonbonded interactions. The candidate aptamers were further analyzed using BLI, with candidate 62 demonstrating the strongest affinity in the sub-micromolar range. This aptamer holds potential for further development to enhance binding efficiency. A primary advantage of this work is its potential applicability to other SARS-CoV-2 variants and possible extension to other viruses.

## Figures and Tables

**Table 1:** List of the IpTM/pTMscore, hydrogen bonds, and other types of interactions predicted in the docked complexes of the top-ranked candidate aptamers (23, 32, and 62) and RBD of the representative variants, BA.2, EG.5, and XBB.1.5, using the AF3 and visualized using the PLIP server.

Variant	Aptamer	IpTM/pTMscore	Hydrogen Bonds	Other Interactions
**BA.2**	Apt23	0.35/0.76	Tyr117 (3.15 Å), Phe158 (2.19 Å), Ser162 (3.02 Å)	***Salt bridges:*** Arg161 (2.84 Å), Arg166 (2.76 Å)
	Apt32	0.51/0.8	Arg71(0.90 Å), Asn73 (2.66 Å), Tyr117 (3.52 Å), Arg166 (3.19 Å), Pro167 (3.06 Å), Tyr169 (2.69 Å), Gly172 (2.87 Å)	***Hydrophobic: *** Arg161 (3.97 Å), Thr168 (3.84 Å) ***π-Stacking: *** His173 (3.81 Å) ***Salt bridges:*** Arg166 (3.46 Å)
	Apt62	0.51/0.8	Tyr89 (3.05 Å), Leu123 (3.54 Å), Phe154 (3.04 Å), Asn155 (2.12, 3.26 Å), Tyr157 (2.96, 3.26 Å), Arg161 (2.95 Å), Arg166 (2.63 Å), Tyr169 (2.25 Å)	***Hydrophobic: *** Tyr169A (3.33 Å), His173 (3.64 Å) ***π-Stacking: *** Phe124 (4.59 Å) ***π-Cation Interactions:*** Arg166 (4.98 Å), His173A (4.05 Å) ***Salt bridges:*** Arg166 (5.37 Å)
**EG.5**	Apt23	0.5/0.76	Tyr117 (2.73 Å), Tyr121 (2.30, 3.06 Å), Gln161 (2.94 Å), Ser162 (1.62, 3.20 Å), Tyr169 (1.96 Å), Hi173 (2.54 Å)	***π-Stacking: *** Tyr117 (3.91 Å), Tyr169 (4.04, 5.33 Å) ***π-Cation interactions: *** Tyr117 (3.71 Å) ***Salt bridges:*** Arg71 (4.46 Å)
	Apt32	0.44/0.74	Arg71 (2.31, 2.47, 2.38, 1.97 Å), Tyr117 (2.74, 2.86 Å), Tyr121 (1.98 Å), Gln161 (2.53 Å), Ser162 (3.44 Å), Gly170 (2.45 Å)	***Hydrophobic: *** Tyr169 (2.82 Å), His173 (3.53 Å) ***π-Cation interactions: *** His173 (4.19 Å) ***Salt bridges:*** Glu74 (4.08 Å)
	Apt62	0.43/0.78	Arg71 (1.88, 2.55 Å), Asn107 (3.31 Å), Lys108 (2.69 Å), Pro113 (1.04 Å), Tyr117 (3.02 Å), Arg166 (2.98 Å), Thr168 (3.11 Å), Val171 (2.42 Å), His173 (2.81 Å), Gln174 (2.38, 2.61 Å)	***Hydrophobic: *** Phe39 (3.25 Å), Asn107 (3.96 Å), Lys108 (3.78 Å) ***π-Stacking: *** Tyr117 (3.85 Å) ***π-Cation interactions: *** Arg166 (4.30 Å) ***Salt bridges:*** Lys46 (5.05 Å), Lys108 (4.80 Å), Arg166 (4.77, 4.90 Å)
**XBB.1.5**	Apt23	0.26/ 0.74	Arg71 (3.25 Å), Arg166 (3.46 Å), Gly172 (1.61 Å)	***π-Stacking:*** His173 (4.87 Å) ***Salt bridges:*** Arg166 (5.37 Å), His173 (5.14 Å)
	Apt32	0.4/0.75	Arg71 (3.55 Å), Ser162 (3.39 Å), Arg166 (3.12 Å), Thr168 (2.38 Å), Tyr169 (2.35 Å), His173 (2.99 Å)	***π-Stacking:*** His173 (3.52, 4.12 Å) ***Salt bridges:*** Arg166 (4.70 Å)
	Apt62	0.38/0.77	Arg71 (3.49 Å), Asn73 (3.16 Å), Asn85 (3.67 Å), Asn105 (2.59 Å), Lys108 (2.84 Å), Tyr121 (2.46 Å), Ser162 (2.58 Å), Arg166 (2.95, 3.18 Å), Thr168 (3.09 Å), Tyr169 (3.35, 3.64 Å), Val171 (3.12 Å), Gly172 (2.64 Å)	***Hydrophobic: *** Asn107 (3.82 Å) ***π-Stacking: *** Phe42 (4.51 Å) ***Salt bridges:*** Lys46 (4.66 Å), Arg71 (5.42 Å), Arg166 (5.44 Å)

**Table 2:** Different binding free energy components (excluding entropic contributions) estimated for candidate aptamers, including binding energy (kJ/mol), van der Waal energy (∆EvdW), electrostatic energy (∆Elec), polar solvation energy (∆G polar), and SASA.

Ligand	Van Der Waals Energy (kJ/mol)	Electrostatic Energy (kJ/mol)	Polar Solvation Energy (kJ/mol)	SASA Energy (kJ/mol)	Binding Energy (kJ/mol)
Aptamer 11	−540.941 ± 32.469	−5240.469 ± 204.737	1184.224 ± 144.540	−55.906 ± 3.279	−4653.091 ± 170.939
Aptamer 22	−405.856 ± 31.896	−5299.972 ± 194.629	845.522 ± 130.716	−39.284 ± 3.265	−4899.591 ± 207.097
Aptamer 23	−472.468 ± 36.798	−6132.275 ± 278.192	1459.135 ± 136.837	−52.844 ± 2.892	−5198.452 ± 263.879
Aptamer 32	−524.211 ± 21.638	−6123.863 ± 152.738	1256.218 ± 135.285	−47.602 ± 2.356	−5439.458 ± 176.559
Aptamer 40	−462.640 ± 35.759	−5638.644 ± 215.213	1352.672 ± 175.234	−47.850 ± 3.213	−4796.462 ± 134.095
Aptamer 62	−407.368 ± 24.506	−6022.193 ± 149.595	1270.769 ± 126.799	−46.032 ± 2.465	−5204.824 ± 156.228

**Table 3:** BLI kinetic constant measurements. Data are represented as mean ± standard deviation (*n* = 2).

Candidate Aptamer	Mean	SD	Mean	SD	Mean	SD
11	4.25 × 10^−7^	1.46 × 10^−7^	4.29 × 10^4^	1.47 × 10^4^	1.72 × 10^−2^	3.54 × 10^−5^
22	5.45 × 10^−6^	1.70 × 10^−6^	4.27 × 10^3^	8.61 × 10^2^	2.25 × 10^−2^	2.57 × 10^−3^
23	2.93 × 10^−5^	1.04 × 10^−5^	9.62 × 10^2^	2.05 × 10^2^	2.71 × 10^−2^	4.04 × 10^−3^
40	5.71 × 10^−7^	1.73 × 10^−7^	3.62 × 10^4^	7.86 × 10^3^	2.00 × 10^−2^	1.78 × 10^−3^
62	2.85 × 10^−7^	1.41 × 10^−7^	4.50 × 10^4^	1.89 × 10^4^	1.15 × 10^−2^	9.33 × 10^−4^

## Data Availability

The original contributions presented in this study are included in the article/supplementary material. Further inquiries can be directed to the corresponding author.

## Supplementary Materials

The following are available online at https://www.mdpi.com/article/10.3390/biom15060805/s1, Figure S1: diagrammatic representation of candidate aptamers highlighting the interaction sites with the SARS-CoV-2 spike protein RBD. Figure S2: 3D structures of six top-ranked candidate aptamers (11, 22, 23, 32, 40, and 62). Table S1: details of binding energy, ligand RMSD, and molecular interactions (hydrogen bonds and nonbonded contacts) of the top-ranked/representative aptamers docking complexes with Spike RBD of SARS-CoV-2 (Wuhan-Hu-1 strain) and Omicron variants (BA.1, BA.2, XBB.1.5, and EG.5). Interactions with mutations are highlighted in bold. File S1: the sequences of the best 83 aptamer candidates.
