# Supplementary material for: Aptamer Development for SARS-CoV-2 and Omicron Variants Using the Spike Protein Receptor Binding Domain as a Potential Diagnostic Tool and Therapeutic Agent
Source: Biomolecules. 2025 Jun 1;15(6):805. doi: 10.3390/biom15060805 (PMC12191217; doi:10.3390/biom15060805)
Supplement: Supplementary file 1 [file biomolecules-15-00805-s001.zip › Supplementary Figure S1.pdf]

Chain A

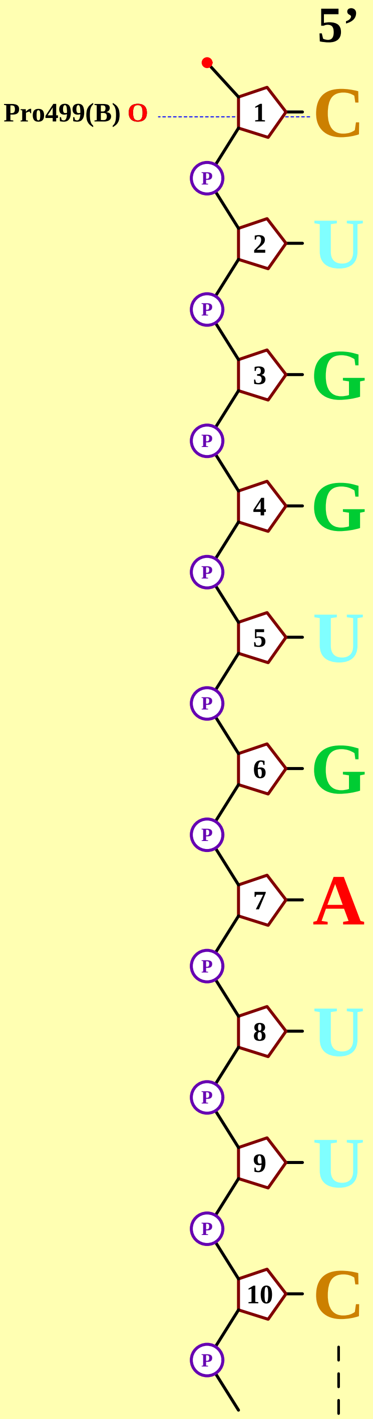

Key

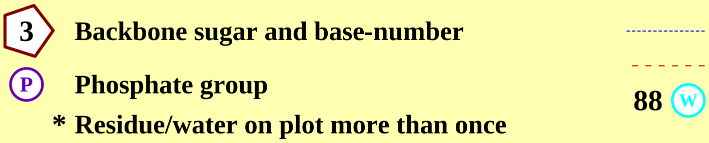

Backbone sugar and base-number

Phosphate group

* Residue/water on plot more than once

Hydrogen bond to DNA

Nonbonded contact to DNA (< 3.35A)

Water molecule and number

file

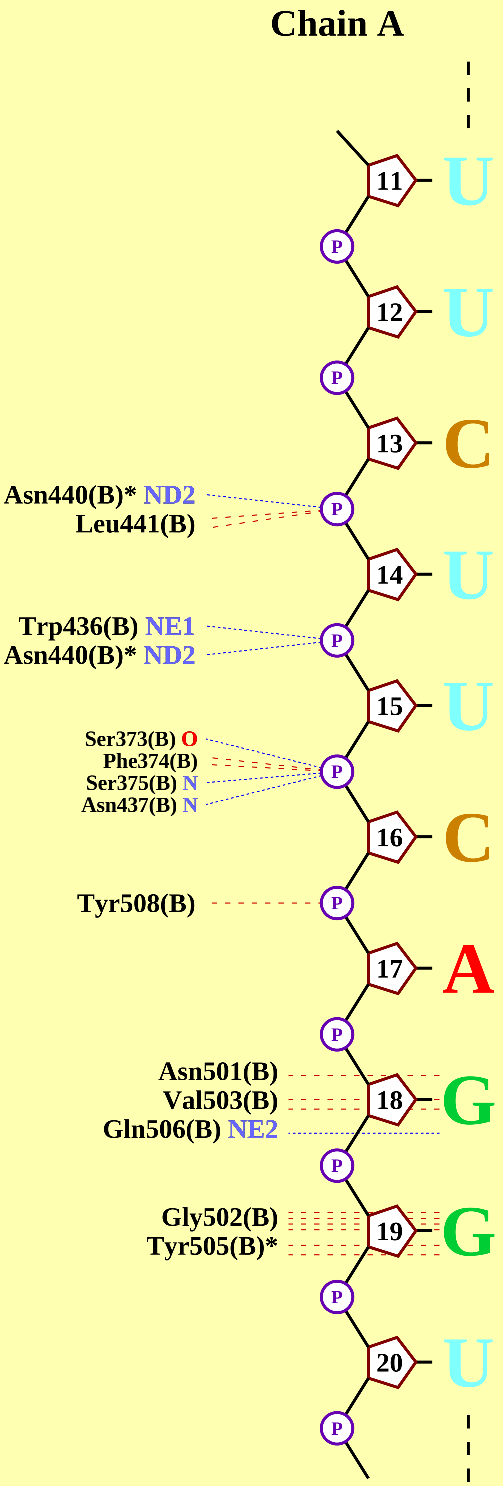
Chain A
11
U
12
U
13
C
Asn440(B)* ND2
Leu441(B)
14
U
Trp436(B) NE1
Asn440(B)* ND2
15
U
Ser373(B) O
Phe374(B)
Ser375(B) N
Asn437(B) N
16
C
Tyr508(B)
17
A
Asn501(B)
Val503(B)
Gln506(B) NE2
18
G
Gly502(B)
Tyr505(B)*
19
G
20
U

Key

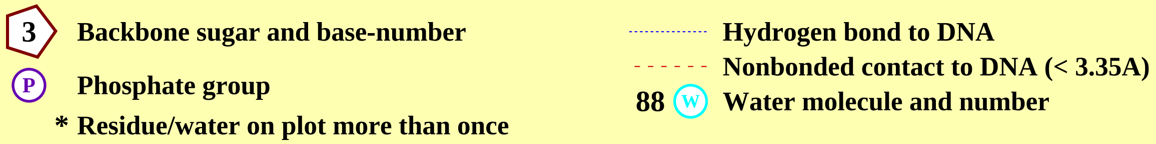
3 Backbone sugar and base-number
Phosphate group
* Residue/water on plot more than once
Hydrogen bond to DNA
Nonbonded contact to DNA (< 3.35A)
88 Water molecule and number

file

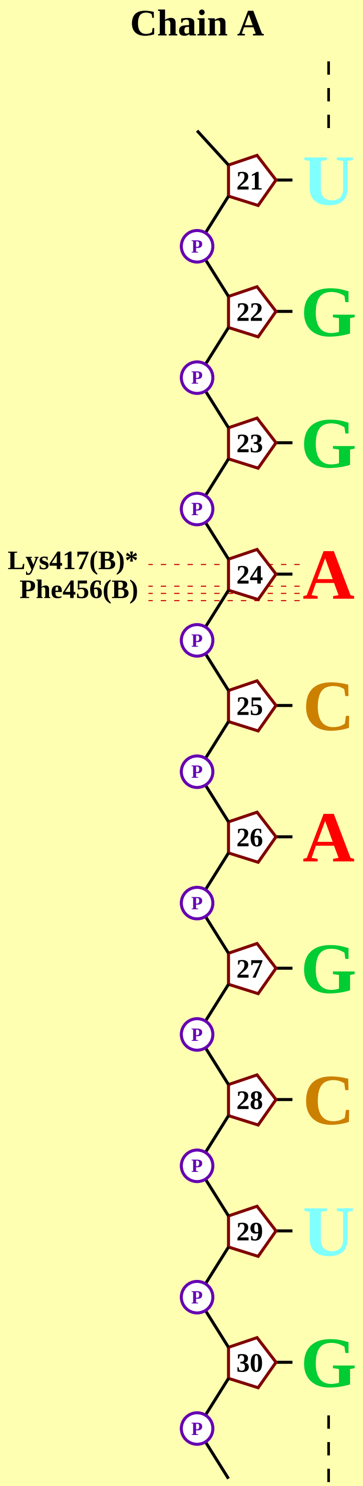

Key

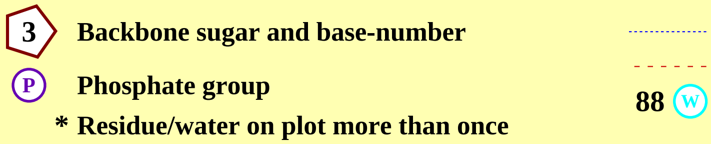

Backbone sugar and base-number

Phosphate group

* Residue/water on plot more than once

Hydrogen bond to DNA

Nonbonded contact to DNA (< 3.35A)

88 Water molecule and number

file

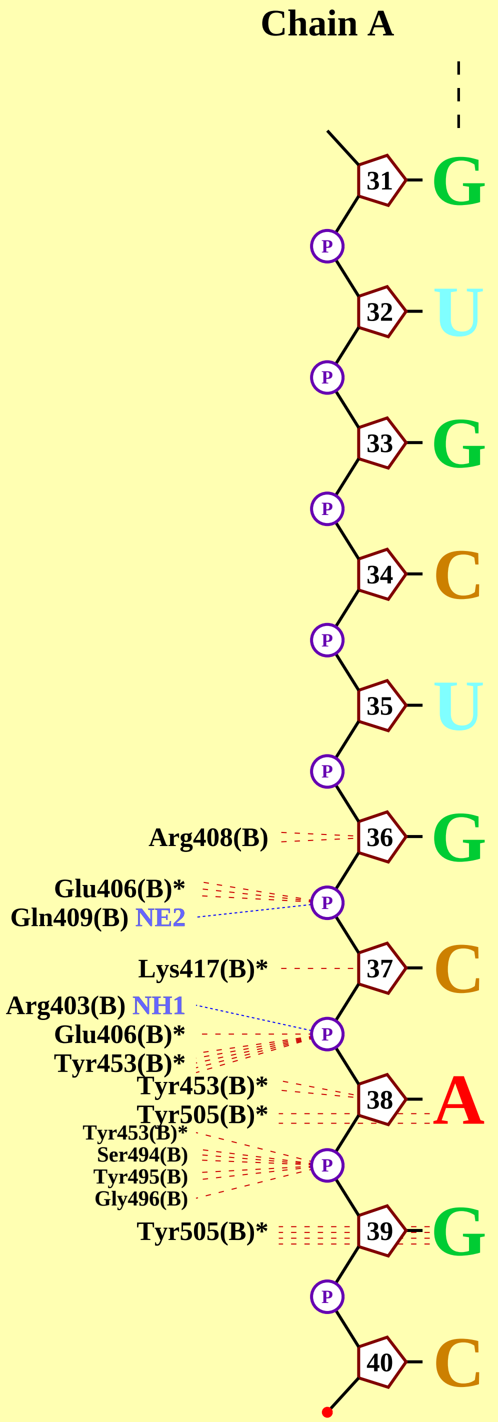

Key

3 Backbone sugar and base-number

P Phosphate group

* Residue/water on plot more than once

Hydrogen bond to DNA

Nonbonded contact to DNA (< 3.35A)

88 W Water molecule and number

file

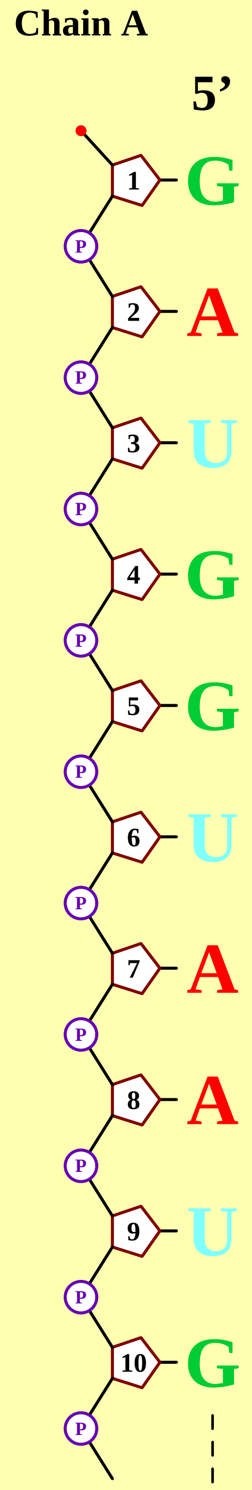

Key

3 Backbone sugar and base-number
P Phosphate group
* Residue/water on plot more than once

Hydrogen bond to DNA
Nonbonded contact to DNA (< 3.35A)
88 W Water molecule and number

file

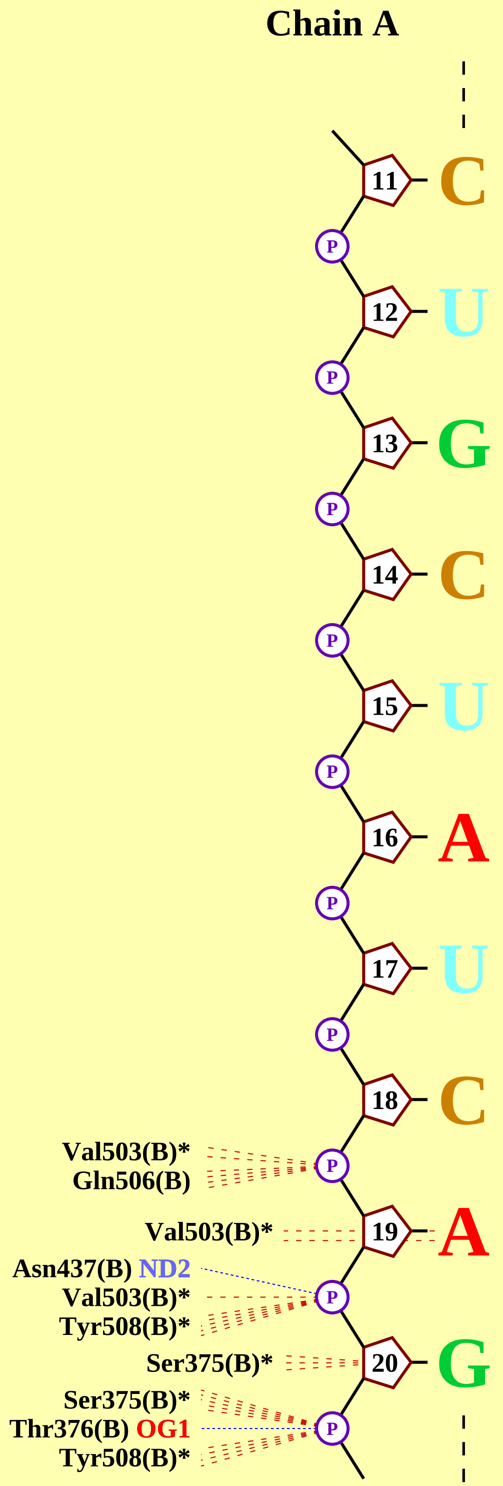

Key

3 Backbone sugar and base-number

P Phosphate group

* Residue/water on plot more than once

Hydrogen bond to DNA

Nonbonded contact to DNA (< 3.35A)

88 W Water molecule and number

file

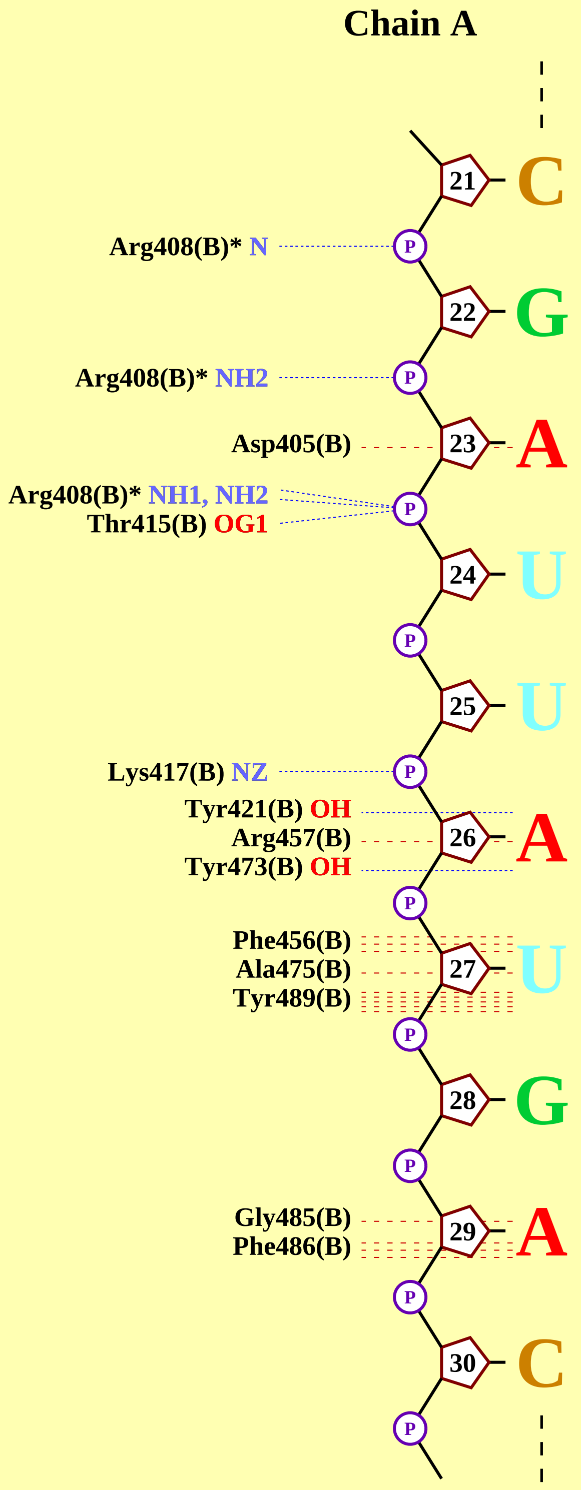

Chain A
21 C
Arg408(B)* N
P
22 G
Arg408(B)* NH2
P
Asp405(B)
23 A
Arg408(B)* NH1, NH2
Thr415(B) OG1
P
24 U
P
25 U
Lys417(B) NZ
P
Tyr421(B) OH
Arg457(B)
Tyr473(B) OH
26 A
P
Phe456(B)
Ala475(B)
Tyr489(B)
27 U
P
28 G
P
Gly485(B)
Phe486(B)
29 A
P
30 C
P

Key

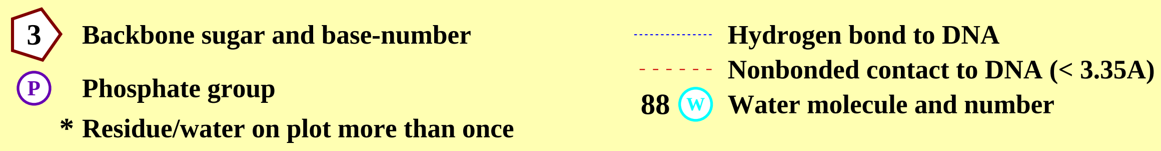

3 Backbone sugar and base-number
P Phosphate group
* Residue/water on plot more than once
Hydrogen bond to DNA
Nonbonded contact to DNA (< 3.35A)
88 W Water molecule and number

file

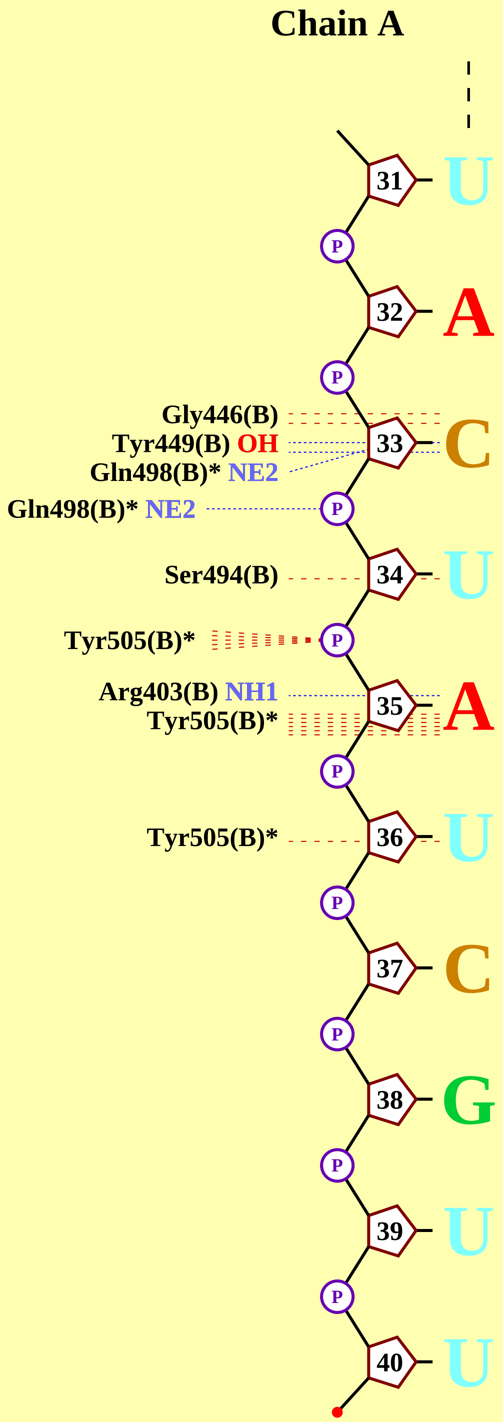
Chain A
31
U
32
A
Gly446(B)
Tyr449(B) OH
Gln498(B)* NE2
33
C
Gln498(B)* NE2
Ser494(B)
34
U
Tyr505(B)*
Arg403(B) NH1
Tyr505(B)*
35
A
Tyr505(B)*
36
U
37
C
38
G
39
U
40
U
P

Key

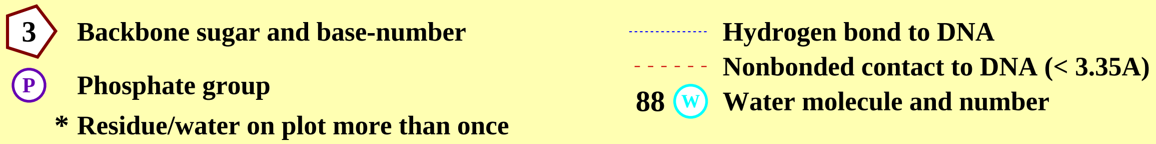
3
Backbone sugar and base-number
P
Phosphate group
* Residue/water on plot more than once
Hydrogen bond to DNA
Nonbonded contact to DNA (< 3.35A)
88
W
Water molecule and number

file

Chain A

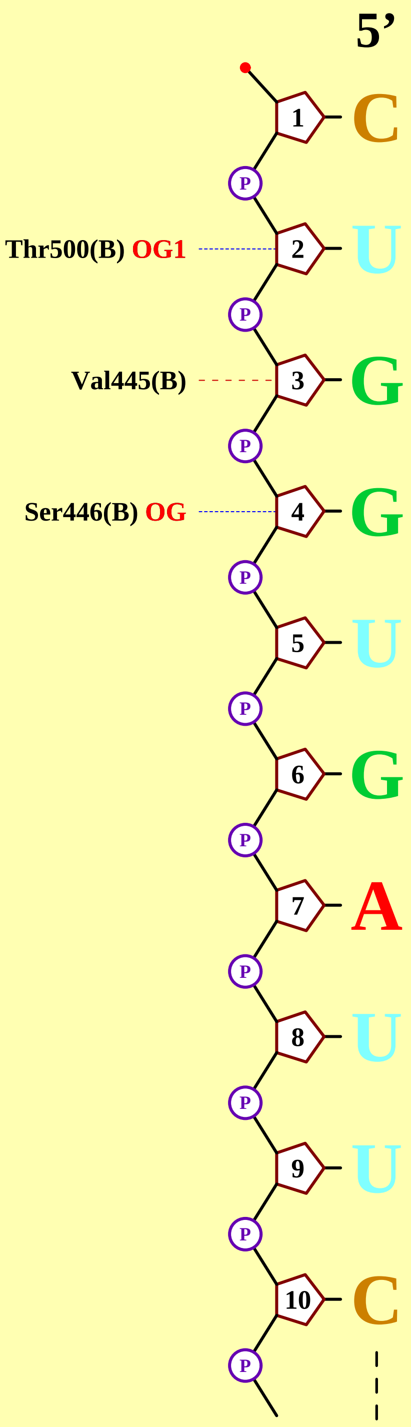

Key

3 Backbone sugar and base-number

P Phosphate group

* Residue/water on plot more than once

Hydrogen bond to DNA

Nonbonded contact to DNA (< 3.35A)

88 W Water molecule and number

file

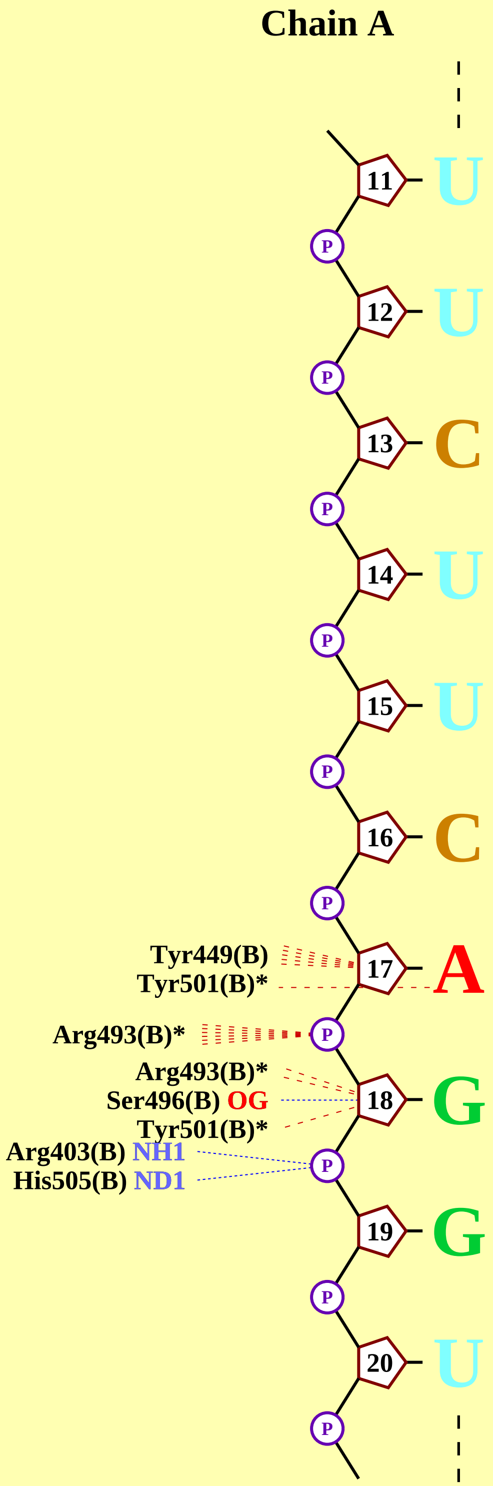
Chain A
11
U
P
12
U
P
13
C
P
14
U
P
15
U
P
16
C
P
Tyr449(B)
Tyr501(B)*
17
A
Arg493(B)*
P
Arg493(B)*
Ser496(B) OG
Tyr501(B)*
18
G
Arg403(B) NH1
His505(B) ND1
P
19
G
P
20
U
P

Key

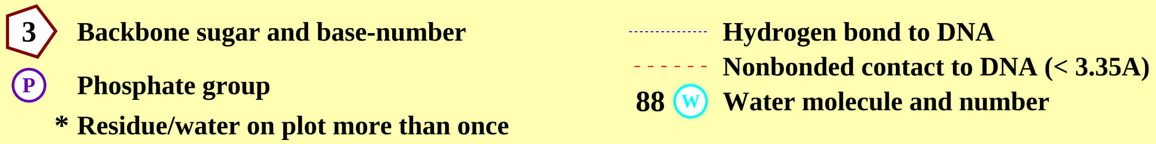
3
Backbone sugar and base-number
P
Phosphate group
* Residue/water on plot more than once
Hydrogen bond to DNA
Nonbonded contact to DNA (< 3.35A)
88
W
Water molecule and number

file

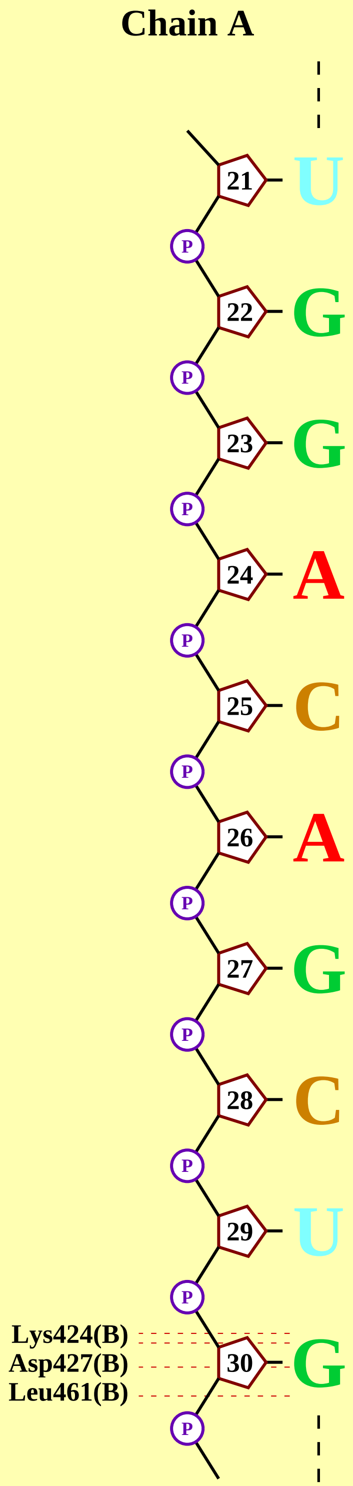

Key

3 Backbone sugar and base-number

P Phosphate group

* Residue/water on plot more than once

Hydrogen bond to DNA

Nonbonded contact to DNA (< 3.35A)

88 W Water molecule and number

file

Chain A

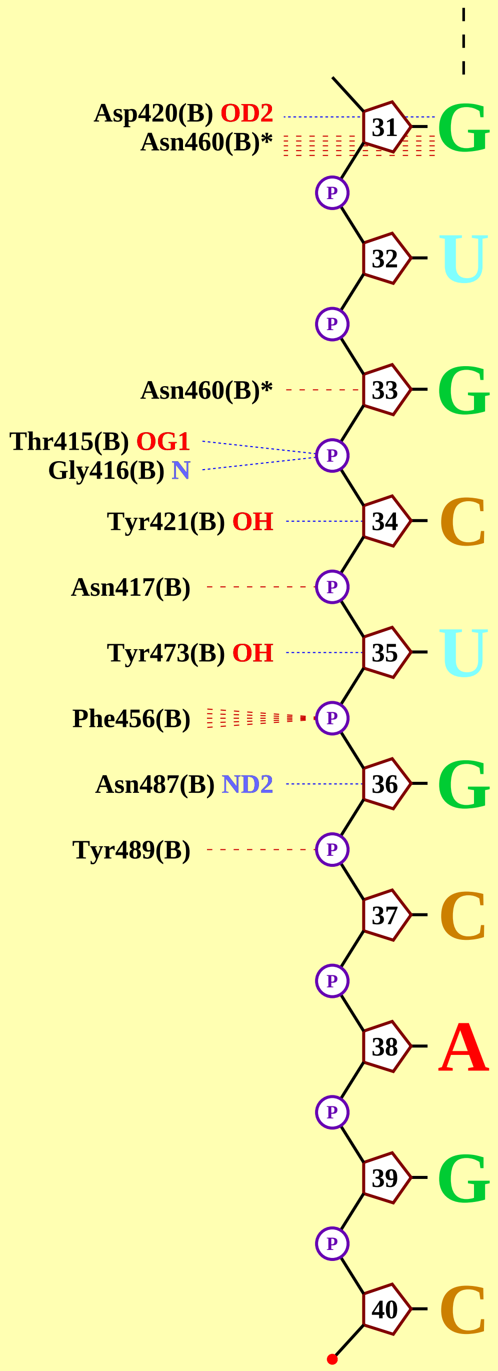

Key

3 Backbone sugar and base-number

P Phosphate group

* Residue/water on plot more than once

Hydrogen bond to DNA

Nonbonded contact to DNA (< 3.35A)

88 W Water molecule and number

file

Chain A

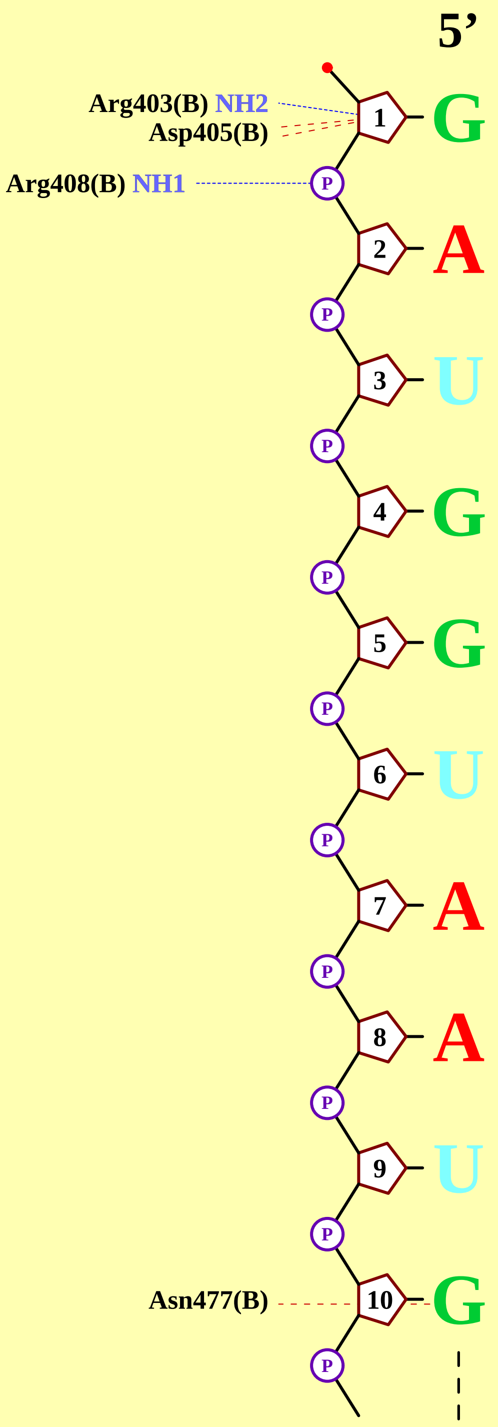

Key

3 Backbone sugar and base-number

P Phosphate group

* Residue/water on plot more than once

Hydrogen bond to DNA

Nonbonded contact to DNA (< 3.35A)

88 W Water molecule and number

file

Chain A

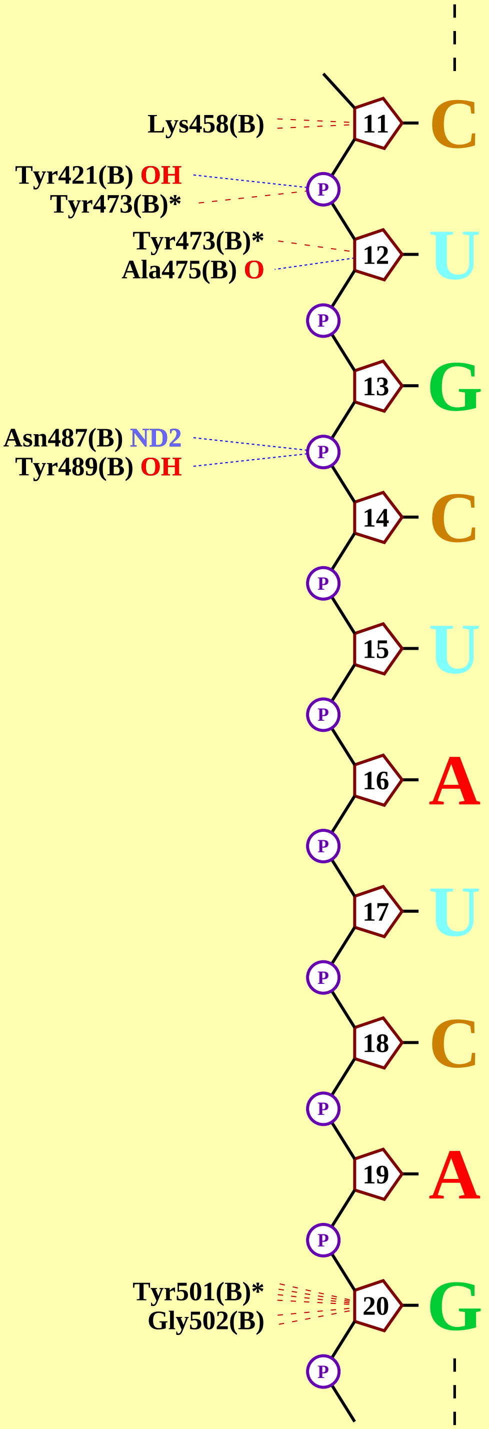

Key

3 Backbone sugar and base-number

P Phosphate group

* Residue/water on plot more than once

Hydrogen bond to DNA

Nonbonded contact to DNA (< 3.35A)

88 W Water molecule and number

file

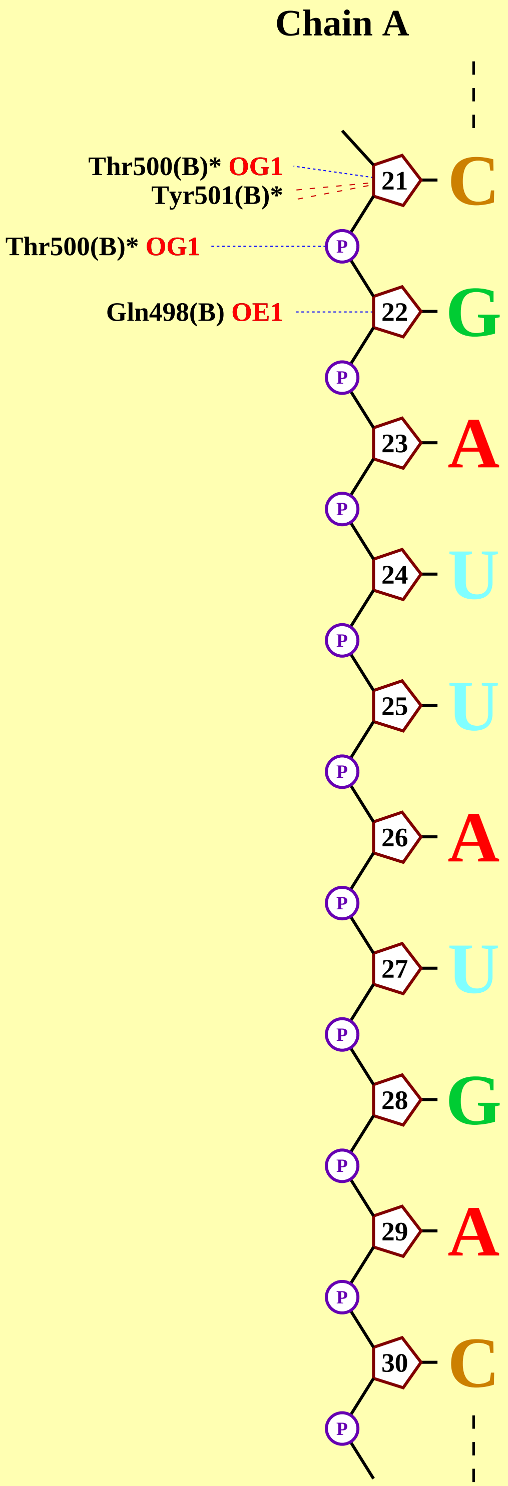

Key

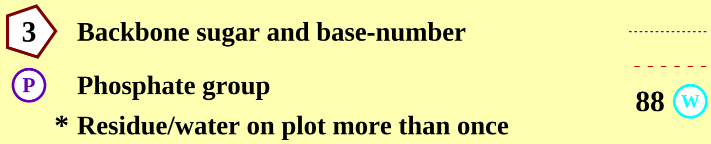

Backbone sugar and base-number

Phosphate group

* Residue/water on plot more than once

Hydrogen bond to DNA

Nonbonded contact to DNA (< 3.35A)

Water molecule and number

file

Chain A

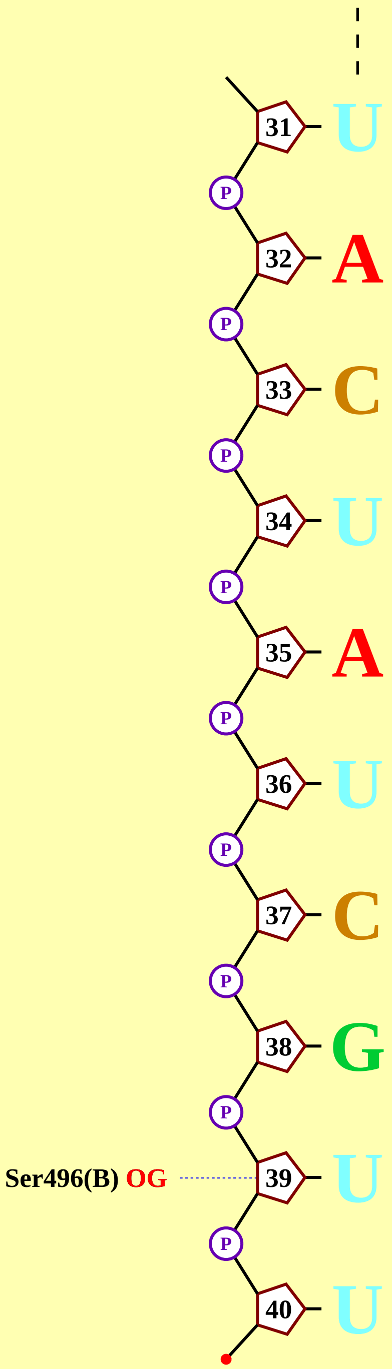

Key

3 Backbone sugar and base-number

P Phosphate group

* Residue/water on plot more than once

Hydrogen bond to DNA

Nonbonded contact to DNA (< 3.35A)

88 W Water molecule and number

file
