# Supplementary material for: Aptamer Development for SARS-CoV-2 and Omicron Variants Using the Spike Protein Receptor Binding Domain as a Potential Diagnostic Tool and Therapeutic Agent
Source: Biomolecules. 2025 Jun 1;15(6):805. doi: 10.3390/biom15060805 (PMC12191217; doi:10.3390/biom15060805)
Supplement: Supplementary file 1 [file biomolecules-15-00805-s001.zip › Supplementary Table S1.pdf]

Supplementary Table:

Table S1. Details of binding energy, ligand RMSD, and molecular interactions (hydrogen bonds and non-bonded contacts) of the top-ranked/representative aptamers docking complexes with Spike RBD of SARS-CoV-2 (Wuhan-Hu-1 strain) and Omicron variants (BA.1, BA.2, XBB.1.5, and EG.5). Interactions with mutations are highlighted in bold.

S.No	Aptamer	Binding energy (kcal/mol)	Ligand RMSD (Å)	Hydrogen bonds	Nonbonded contact (< 3.35 Å)
**Top-ranked aptamers SARS-CoV-2 (Wuhan-Hu-1 strain)**					
1.	Aptamer 11	-325.15	37.52	Asp405, Arg408, Thr415	Ser375, Lys378, Arg403, Phe456, Tyr473, Gly485, Phe486, Tyr489, Gln493
2.	Aptamer 22	-328.81	45.85	Thr385, Tyr369, Cys379, Gln414, Thr415	Lys378, Tyr380, Lys378, Pro384, Arg403, Glu406, Gly413, Tyr453, Leu455, Phe456, Phe486, Gly485, Tyr489
3.	Aptamer 23	-328.46	24.76	Ser373, Asn437, Arg408	Ala372, Lys378, Phe374, Ser375, Thr376, Arg408, Gln414, Thr415, Asn440, Tyr505
4.	Aptamer 32	-318.28	29.62	Ser373, Ser375, Trp436, Arg403, Gln409, Asn437, Asn440, Pro499, Gln506	Phe374, Glu406, Arg408, Lys417, Leu441, Tyr453, Phe456, Ser494, Tyr495, Gly496, Tyr508, Asn501, Gly502, Val503, Tyr505
5.	Aptamer 40	-318.57	35.89	Arg403, Tyr421, Tyr453, Tyr473, Gln493, Thr500, Gly502, Tyr505	Arg403, Lys417, Leu455, Phe456, Arg457, Ala475, Tyr489, Gln498, Asn501

6.	Aptamer 62	-326.89	21.24	Arg408, Tyr421, Lys417, Asn437, Tyr473, Thr376, Gln498, Tyr449, Tyr508	Ser375, Arg403, Asp405, Thr415, Gly446, Phe456, Arg457, Ala475, Gly485, Phe486, Tyr489, Ser494, Val503, Tyr505, Gln506, Tyr508
**Top-ranked aptamers BA.1**					
1.	Aptamer 11	-315.31	42.05	Thr415, **Arg493**	Arg403, Gly413, Gln414, Asp427, Leu455, Phe456, Asn460, **Ala484**, Gly485, Phe486, Tyr489
2.	Aptamer 22	-301.03	40.40	Arg408, Phe490, **Arg493**	Arg403, Asp405, **Asn417**, **Ala484**, Gly485, Cys488, Tyr489, Leu492, **Tyr501**, **His505**
3.	Aptamer 23	-353.61	40.52	Thr415, **Asn417**, Gly447, **Arg493**, Ser494, **Ser496**, **Tyr501**	Arg403, Gly413, Lys444, Tyr449, Leu455, Phe490, Leu492, **Gln498**, **His505**
4.	Aptamer 32	-335.38	36.29	Arg403, Thr415, Gly416, Asp420, Tyr421, **Ser446**, Tyr473, Asn487, **Ser496**, Thr500, **His505**	**Asn417**, Lys424, Asp427, Val445, Tyr449, Phe456, Asn460, Leu461, Tyr489, **Arg493**, **Tyr501**
5.	Aptamer 40	-319.62	31.40	Arg403, Asp405, Arg408, Gln409, Thr415, **Asn417**, **Asn477**, Asn487	Gln414, Gly416, Asp420, Ala475, Gly476, Phe486, Tyr489

6.	Aptamer 62	-328.81	46.90	Arg403, Arg408, Tyr421, Ala475, Asn487, Tyr489, **Ser496**, **Gln498**, Thr500	Asp405, **Asn477**, Lys458, Tyr473, **Tyr501**, Gly502
**Representative aptamers against BA.2**					
1.	Aptamer 23	-343.60	0.70	Thr415, **Asn417**, Tyr449, **Arg493**, Ser494, **Tyr501**	Arg403, Lys444, Gly447, Leu455, Phe456, Asn460, Tyr473, Tyr489, Phe490, **His505**
2.	Aptamer 32	-354.38	2.79	Arg403, Gly416, Asp420, Tyr421, Tyr449, Tyr473, Asn487	Thr415, Lys424, Asp427, Gly446, Phe456, Asn460, Ala475, Tyr489, **Arg493**, Gly496, **Arg498**
3.	Aptamer 62	-304.50	21.86	Arg403, Tyr421, Tyr449, Tyr489, **Arg493**, **Arg498**, Thr500, **Tyr501**, **His505**	**Asn477**, Phe456, Ser494, Gly496
**Representative aptamers against XBB.1.5**					
1.	Aptamer 23	-314.36	57.76	Thr333, Pro337, **His339**, **Lys440**, Asn343	Leu335, Asn360, Val362, **Phe371**, Ala372, **Phe375**, Trp436, Asn437, Thr523, Pro527
2.	Aptamer 32	-320.03	29.64	Tyr449, Gly482, **Ser490**, Gln493	Arg403, Leu452, Tyr453, Ile472, Val483, **Ala484**, Gly485, Tyr489, Ser494, Gly496, **Tyr501**
3.	Aptamer 62	-295.69	22.48	Tyr449, Tyr489, Gly496,	Arg403, Tyr421, **Asn477**, Phe456, Ser494, **His505**

				**Arg498**, Thr500, **Tyr501**	
**Representative aptamers against EG.5**					
1.	Aptamer 23	-297.91	38.53	Arg403, **Asn405**, Gly485, **Ser490**, **Arg498**	**Ser446**, Tyr449, Val483, **Ala484**, **Pro486**, Tyr489, Gln493, **Arg498**, **Tyr501**, **His505**
2.	Aptamer 32	-318.34	29.37	Arg403, Tyr449, **Ser490**, Gln493	Tyr453, Gly482, Val483, Ala484, Gly485, Tyr489, Leu452, Ser494, **Tyr501**
3.	Aptamer 62	-283.65	34.79	Lys378, Cys379, Gly381, Ser383, Gln414, Asp427	**Phe375**, Phe377, Tyr380, Val382, Pro384, **Asn405**, **Tyr501**, Val503, Gly504

Note: Omicron variant (B.1.19) mutations: G339D, S371L, S373P, S375F, N440K, G446S, T478K, G496S, Q498R, K417N, S477N, E484A, Q493R, N501Y, and Y505H.
