# Supplementary figures and images for: Aptamer Development for SARS-CoV-2 and Omicron Variants Using the Spike Protein Receptor Binding Domain as a Potential Diagnostic Tool and Therapeutic Agent
Source: Biomolecules. 2025 Jun 1;15(6):805. doi: 10.3390/biom15060805 (PMC12191217; doi:10.3390/biom15060805)

### 3D structures of top six aptamers (11, 22, 23, 32, 40, and 62)

**Aptamer 11:**

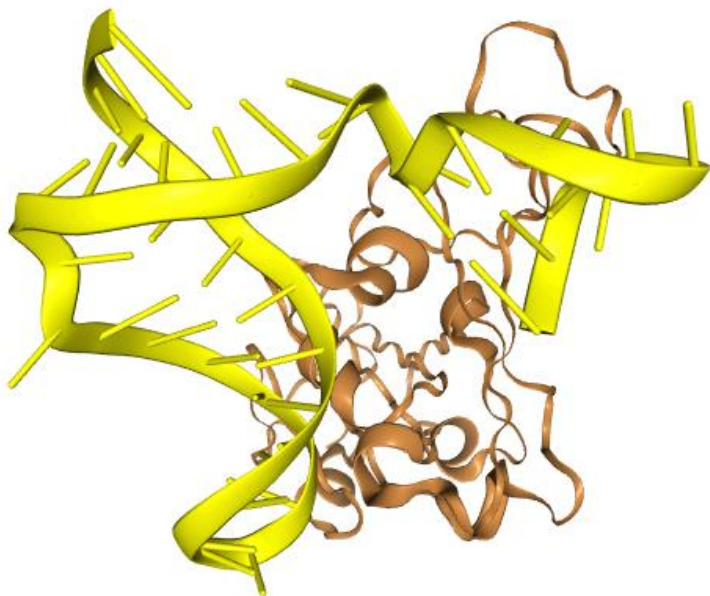

**Aptamer 22:**

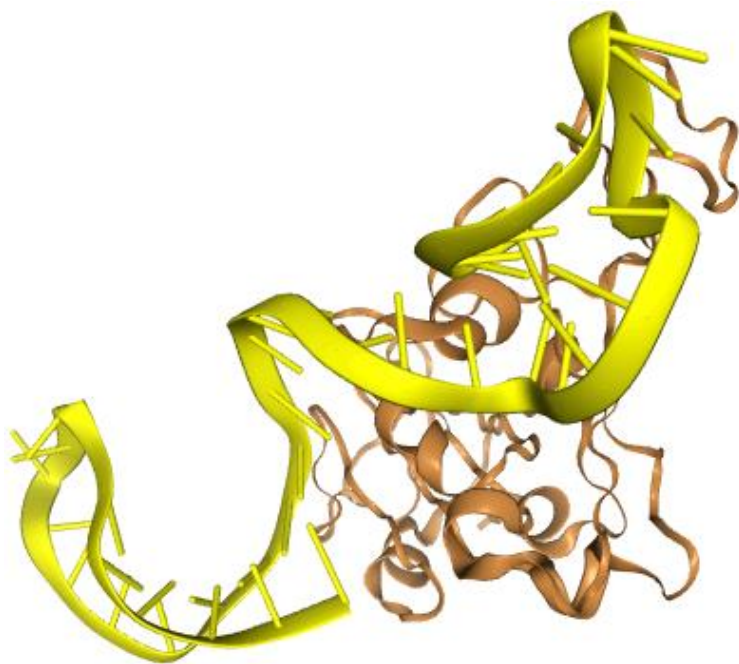

**Aptamer 23:**

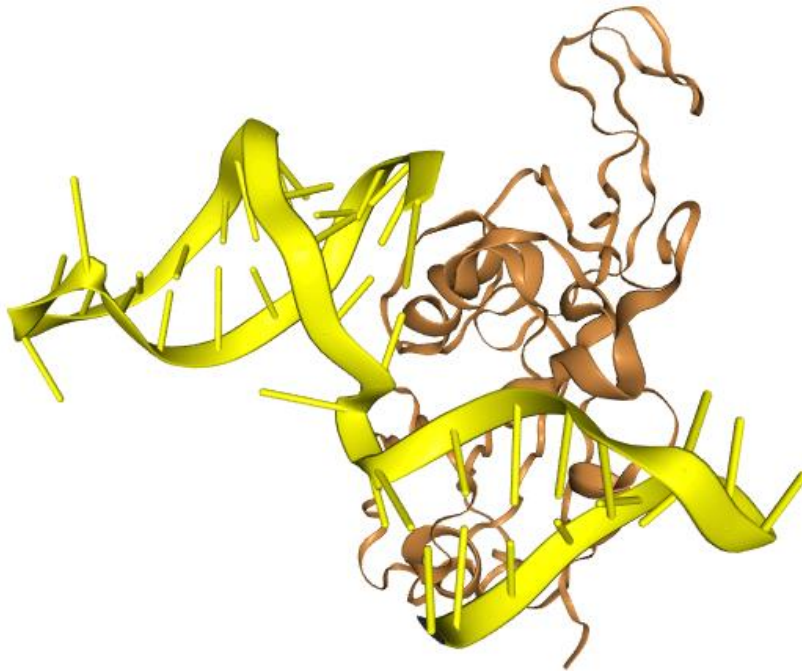

**Aptamer 32:**

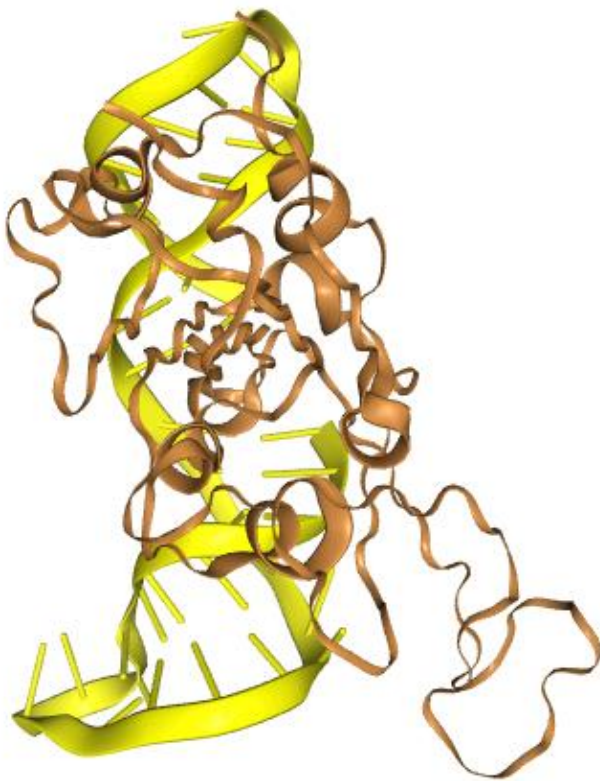

**Aptamer 40:**

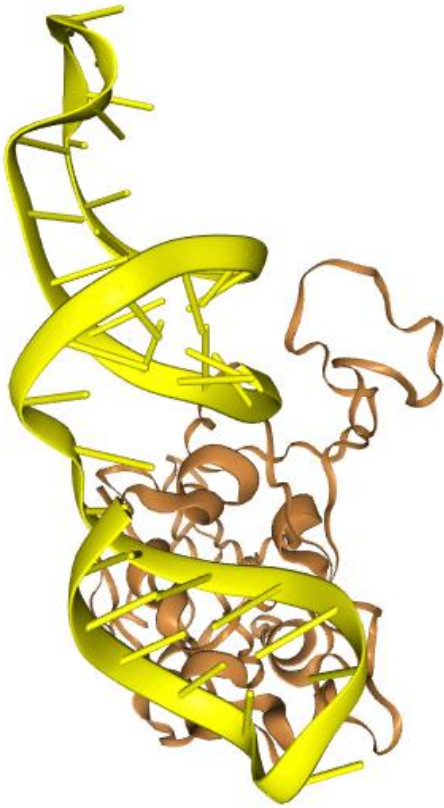

**Aptamer 62:**

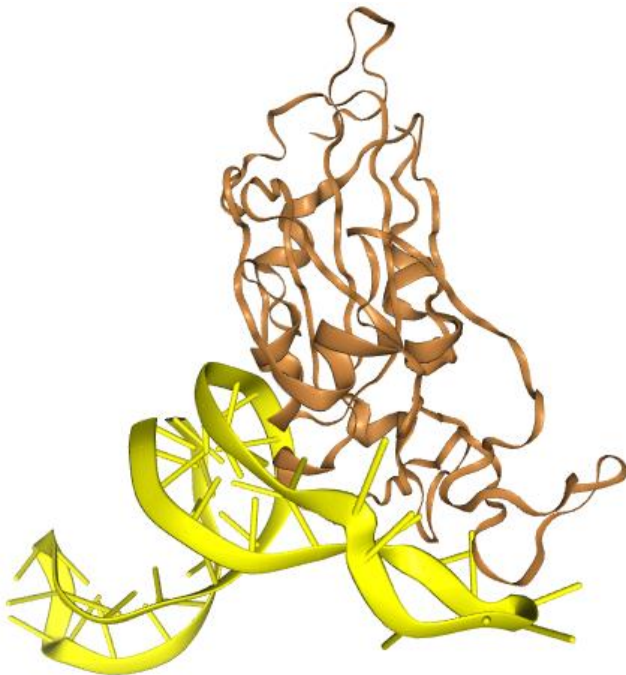

Supplement: Supplementary file 1 [file biomolecules-15-00805-s001.zip › Supplementary Figure S2.pdf]
